# Supplementary material for: Targeting of the m6A eraser ALKBH5 suppresses stemness and chemoresistance of colorectal cancer
Source: Nat Commun. 2025 Dec 13;17:803. doi: 10.1038/s41467-025-67502-0 (PMC12824147; doi:10.1038/s41467-025-67502-0)
Supplement: Supplementary file 1 — Supplementary Information [file 41467_2025_67502_MOESM1_ESM.pdf]

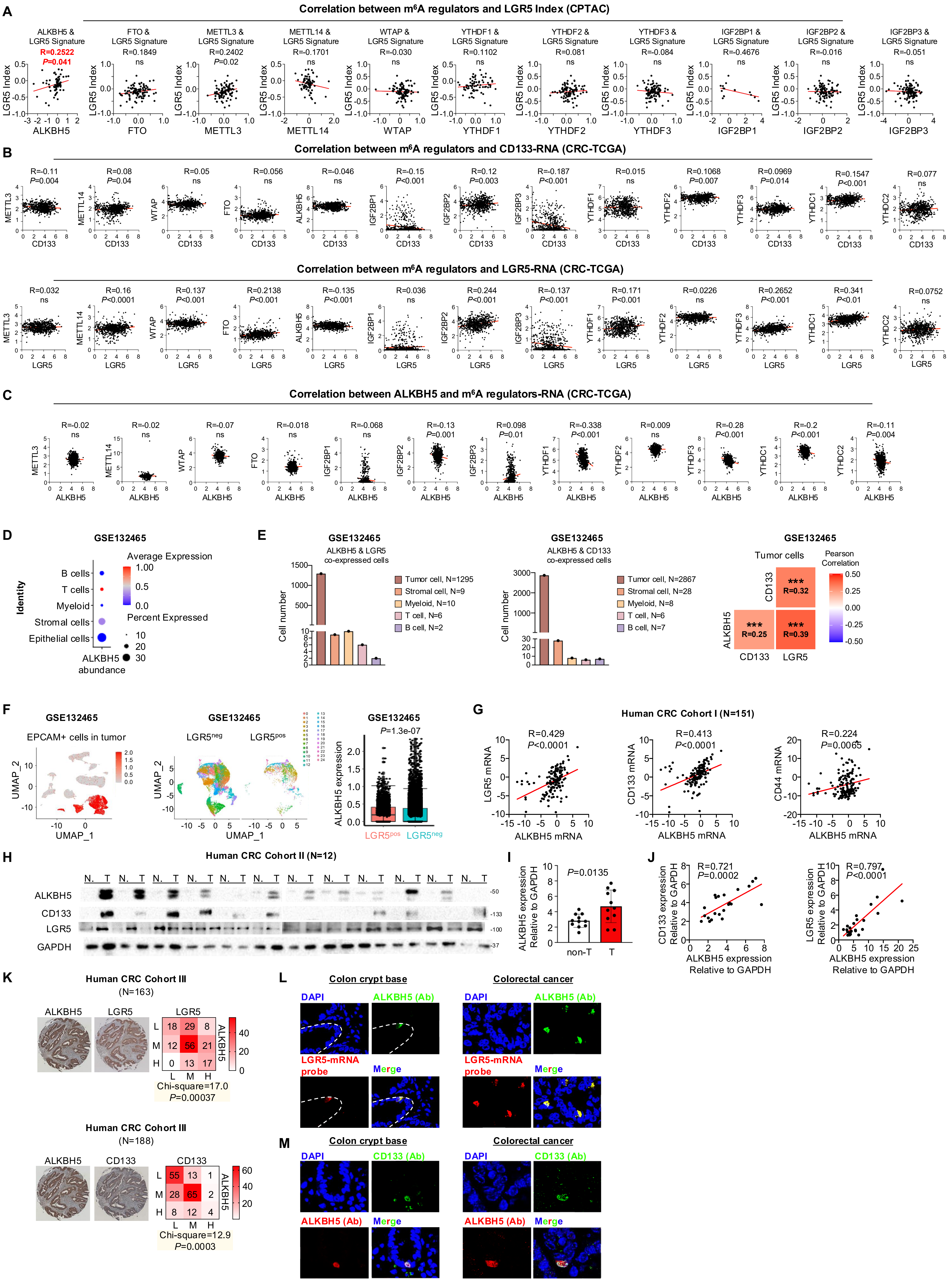

Figure S1: ALKBH5 positively correlated with LGR5 and other stemness markers in CRC patients.

(**A**) Correlation between m^{6}A regulators and LGR5 at protein level (Cohort number: PDC000116).**(B)** Correlation between m^{6}A regulators and stemness markers (CD133 & LGR5) at mRNA level in TCGA-CRC dataset (n=638). **(C)** Correlation between m^{6}A regulators and ALKBH5 at mRNA level in TCGA-CRC cohort (n=638). **(D)** ALKBH5 mRNA expression distribution within CRC microenvironment (GSE132465). **(E)** Correlation between ALKBH5 and stemness markers (CD133 & LGR5) at mRNA level within CRC microenvironment (GSE132465). **(F)** Isolation of epithelial cell in tumor part (left panel); Separation of LGR5^{neg} and LGR5^{pos} epithelial tumor cells (middle panel); Expression of ALKBH5 in LGR5^{neg} and LGR5^{pos} tumor cells in CRC (right panel) (GSE132465). **(G)** Correlation between ALKBH5 with LGR5 (left panel), CD133 (middle panel) and CD44 (right panel) at mRNA level in in-house CRC cohort I (n=151). **(H)** ALKBH5, CD133 and LGR5 expression as determined by western blot in in-house CRC cohort II (n=12). **(I)** Densitometry quantification of ALKBH5 expression in in-house CRC cohort II. (n=11, each dot represents an independent patient) **(J)** Correlation between ALKBH5 and CD133 (left panel), and between ALKBH5 and LGR5 (right panel) in in-house CRC cohort II (n=23, each dot represents an independent sample). **(K)** Correlation between ALKBH5 with LGR5 (n=163) (upper panel) and CD133 (n=188) (lower panel) at protein level in CRC tissue microarrays in in-house CRC cohort III. **(L)** Co immunofluorescence staining of ALKBH5 (anti-ALKBH5 antibody) and LGR5 (anti-LGR5 mRNA probe) in colon crypt base (left panel) and CRC (right panel) in mice. **(M)** Co immunofluorescence staining of ALKBH5 (anti-ALKBH5 antibody) and CD133 (anti-CD133 antibody) in colon crypt base (left panel) and CRC (right panel) in mouse.

Results are presented as mean ± S.D. Each spot represents one subject. Statistical significance was determined by paired samples t-test.

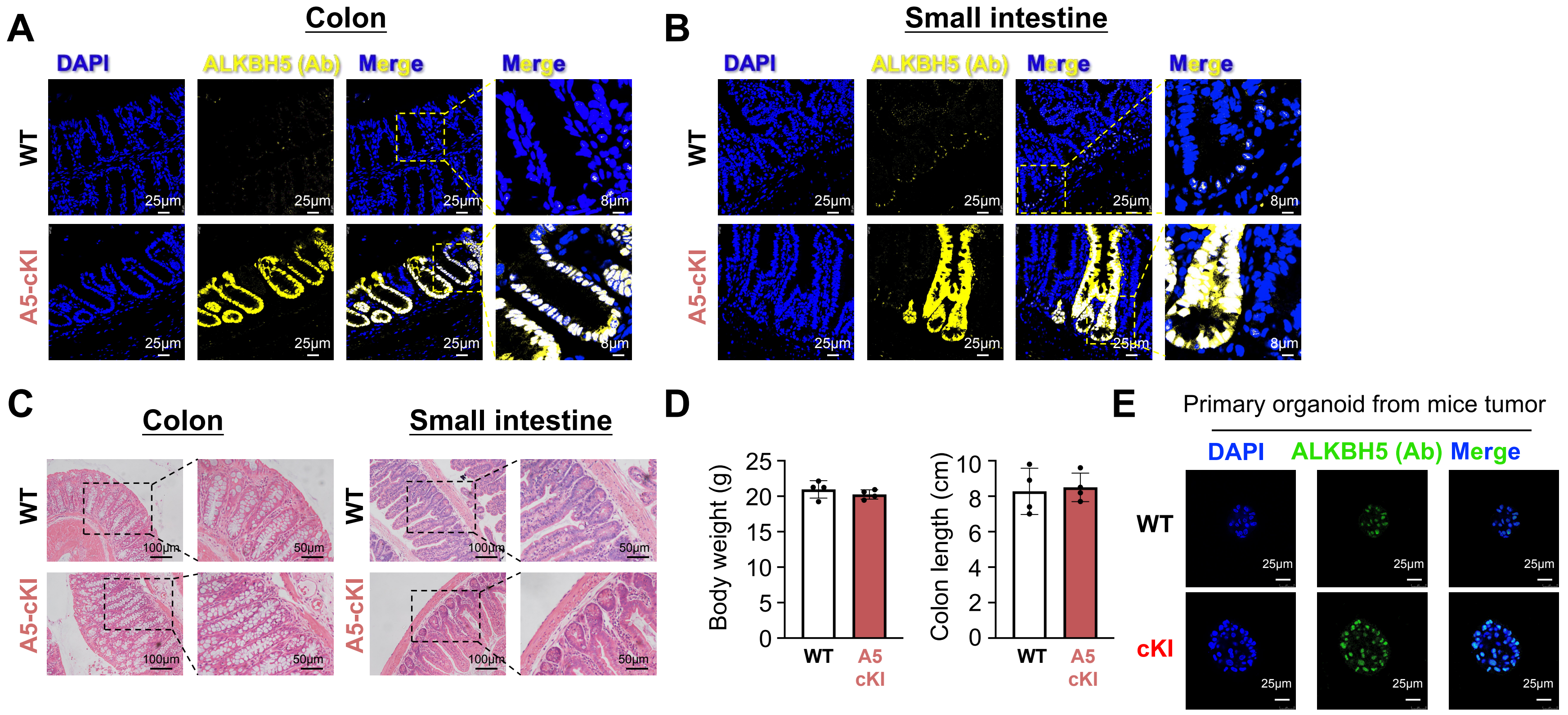

Figure S2: ALKBH5's role in long-term intestine homeostasis of WT and A5-cKI mice.
(A) Immunofluorescence staining of ALKBH5 (anti-ALKBH5 antibody) in colon of WT mice and A5-cKI mice. **(B)** Immunofluorescence staining of ALKBH5 (anti-ALKBH5 antibody) in small intestine of WT mice and A5-cKI mice. **(C)** H&E staining in colon (left panel) and small intestine (right panel) of WT mice and A5-cKI mice. **(D)** Body weight and colon length of WT mice and A5-cKI mice (n=4, each dot represents an independent mouse). **(E)** Immunofluorescence staining of ALKBH5 (anti-ALKBH5 antibody) in CRC organoids from WT mice and A5-cKI mice.
Results are presented as mean ± S.D. Each spot represents one subject. Statistical significance was determined by paired samples t-test.

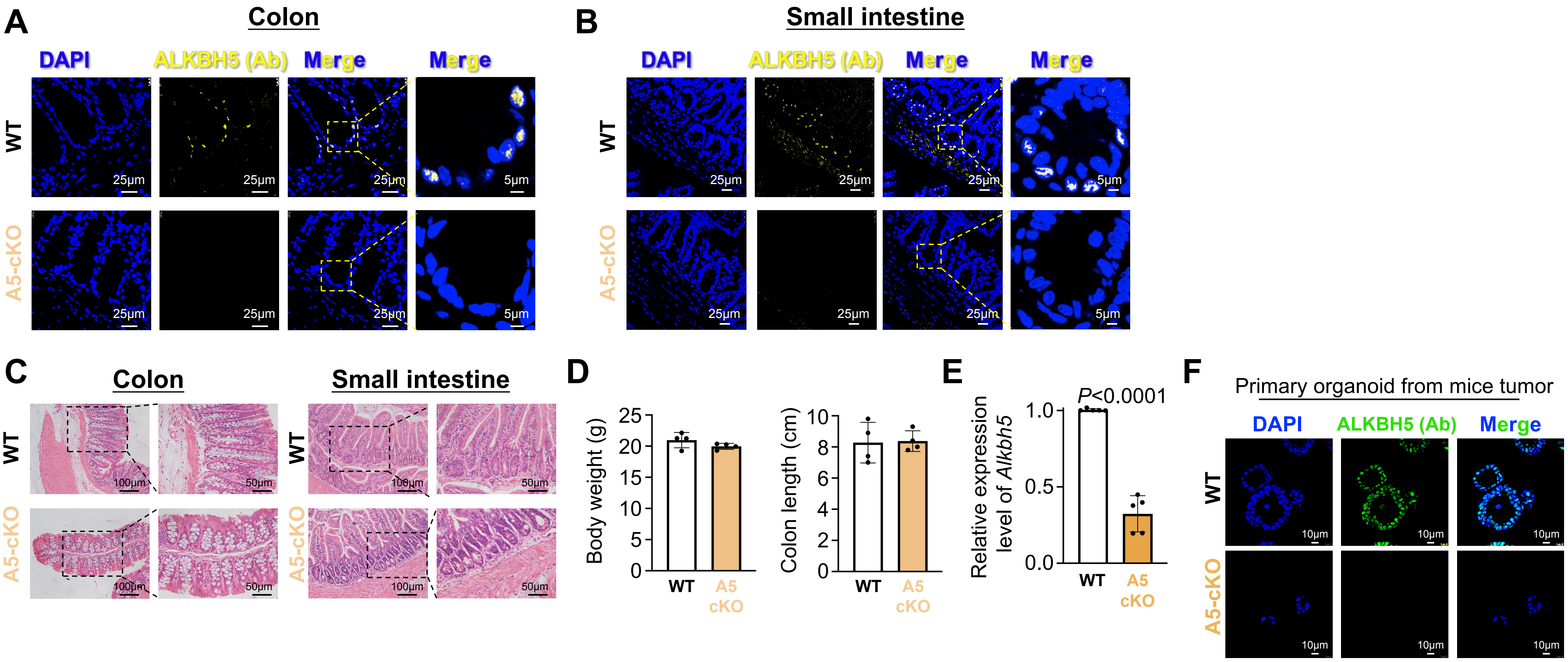

Figure S3: ALKBH5's role in long-term intestine homeostasis of WT and A5-cKO mice.
(A) Immunofluorescence staining of ALKBH5 (anti-ALKBH5 antibody) in colon of WT mice and A5-cKO mice. **(B)** Immunofluorescence staining of ALKBH5 (anti-ALKBH5 antibody) in small intestine of WT mice and A5-cKO mice. **(C)** H&E staining in colon (left panel) and small intestine (right panel) of WT mice and A5-cKO mice. **(D)** Body weight and colon length of WT mice and A5-cKO mice (n=4, each dot represents an independent mouse). **(E)** Alkbh5 mRNA abundance in CRC of WT mice and A5-cKO mice as determined by RT-qPCR (n=5, each dot represents an independent mouse). **(F)** Immunofluorescence staining of ALKBH5 (anti-ALKBH5 antibody) in CRC organoids from WT mice and A5-cKO mice.
Results are presented as mean ± S.D. Each spot represents one subject. Statistical significance was determined by paired samples t-test.

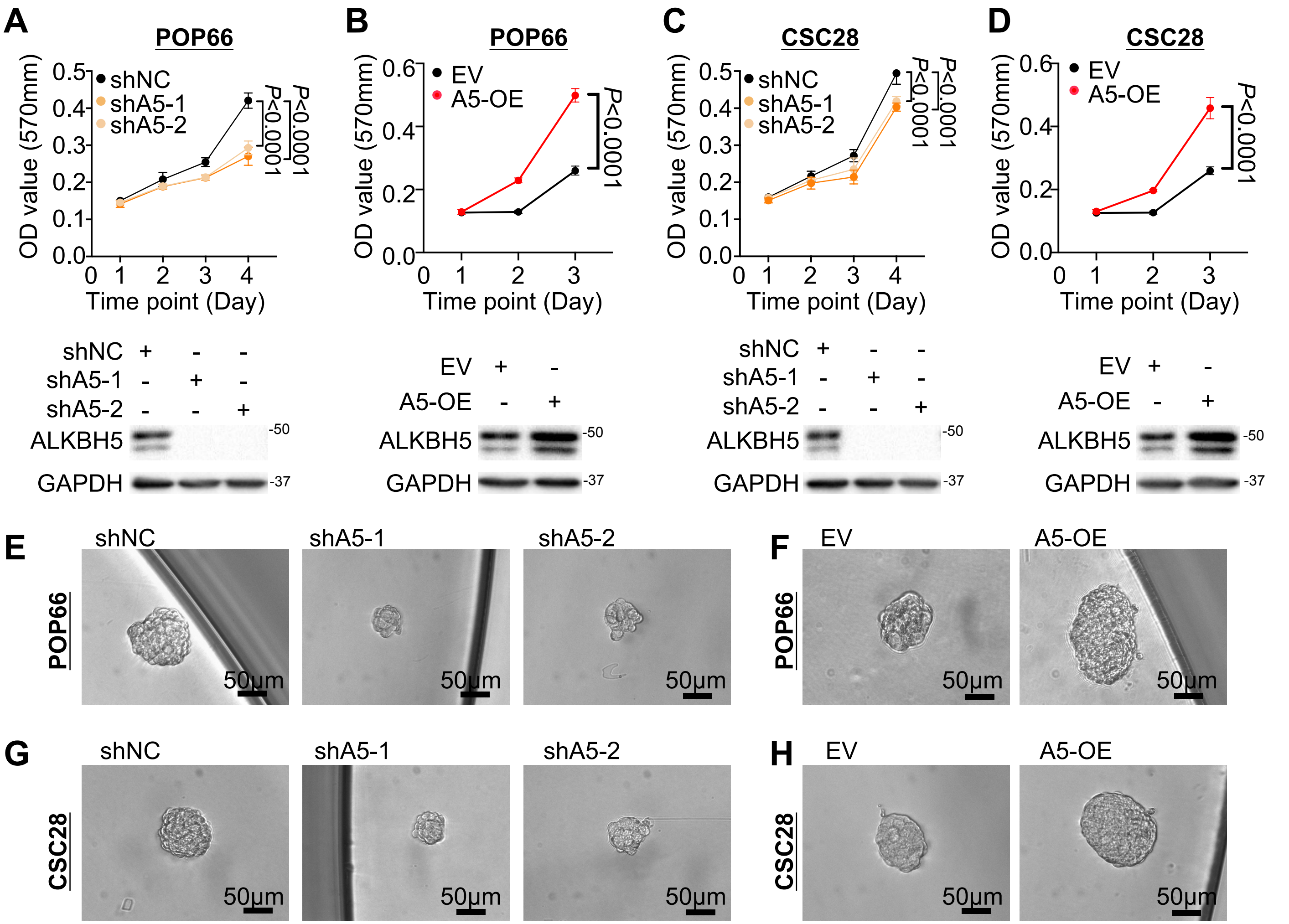

Figure S4. ALKBH5 is essential for CSC proliferation and self-renewal.

(A-D) Function of ALKBH5 in CSC tumor sphere proliferation, as determined by MTT assay (upper) (n=10 for **A**, n=9 for **B**, n=10 for **C**, n=9 for **D**, each dot represents an independent sample); ALKBH5 expression as determined by western-blot (lower). **(E-H)** Representative images of CSC tumor spheres in *in vitro* LDA with over-expression or knockdown of ALKBH5.

Results are presented as mean ± S.D. Each spot represents one subject. Statistical significance was determined by student's *t* test or one-way ANOVA where appropriate.

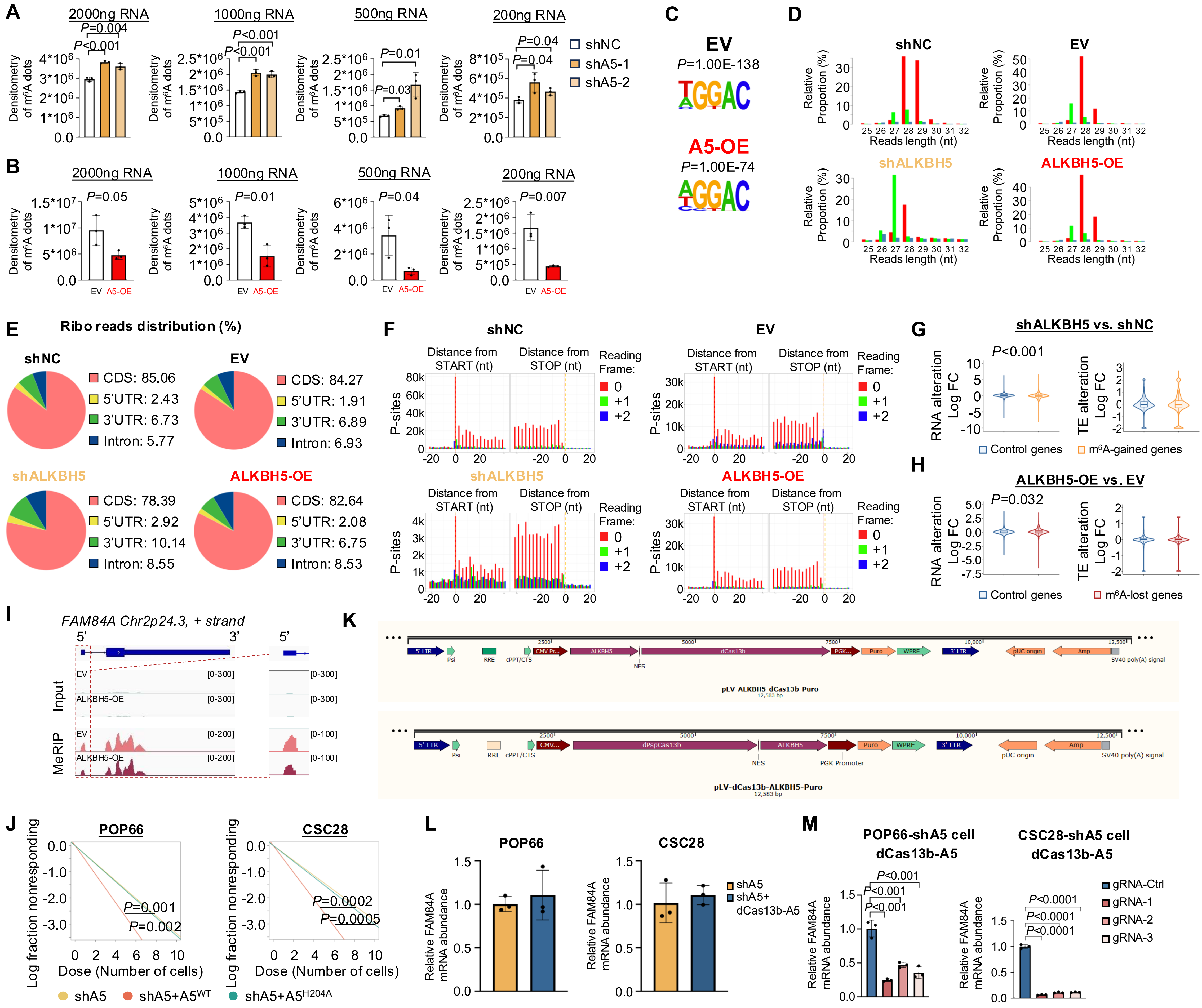

Figure S5. Demethylation role of ALKBH5.

(A) Densitometry quantification of m^6A dots in shNC cells versus shALKBH5 cells across 2000ng RNA, 1000ng RNA, 500ng RNA and 200ng RNA (n=3, each dot represents an independent sample). **(B)** Densitometry quantification of m^6A dots in EV cells versus ALKBH5-OE cells across 2000ng RNA, 1000ng RNA, 500ng RNA and 200ng RNA (n=3, each dot represents an independent sample). **(C)** The top enriched motifs based on m^6A peaks in ALKBH5-overexpressing CSC28 cells and control cells. **(D)** Read-length distribution of ribo-seq in CSC-shNC, CSC-shALKBH5, CSC-EV and ALKBH5-OE cells. **(E)** Percentage of ribo-seq reads mapping to the 5'UTR, coding sequence (CDS), 3'UTR and intron in CSC-shNC, CSC-shALKBH5, CSC-EV and ALKBH5-OE cells. **(F)** The 3-nt periodicity analysis and ribosome-protected frames (RPF) coverage around START and STOP codons of ribo-seq in CSC-shNC, CSC-shALKBH5, CSC-EV and ALKBH5-OE cells. **(G)** RNA expression (left panel) and translation efficiency (right panel) of m^6A-gained genes in CSC-shALKBH5 cells compared with CSC-shNC cells with other genes as control. **(H)** RNA expression (left panel) and translation efficiency (right panel) of m^6A-loss genes in CSC-ALKBH5-OE cells compared with CSC-EV cells with other genes as control. **(I)** Bigwig snapshots of MeRIP-seq reads of FAM84A in ALKBH5-overexpression compared with EV cells. Normalized read densities to input. **(J)** Effect of CSC self-renewal with $ALKBH5^{WT}$ or $ALKBH5^{H204A}$ overexpression as demonstrated by *in vitro* LDA in POP66 and CSC28. **(K)** Diagram of ALKBH5-dCas13b plasmids. **(L)** FAM84A mRNA levels in dCas13b-ALKBH5-transfected colorectal CSCs (n=3, each dot represents an independent sample). **(M)** FAM84A mRNA in colorectal CSCs co-transfected with dCas13b-ALKBH5 and the gRNAs (n=3, each dot represents an independent sample).

Results are presented as mean ± S.D. Each spot represents one subject. Statistical significance was determined by student's t test or one-way ANOVA where appropriate.

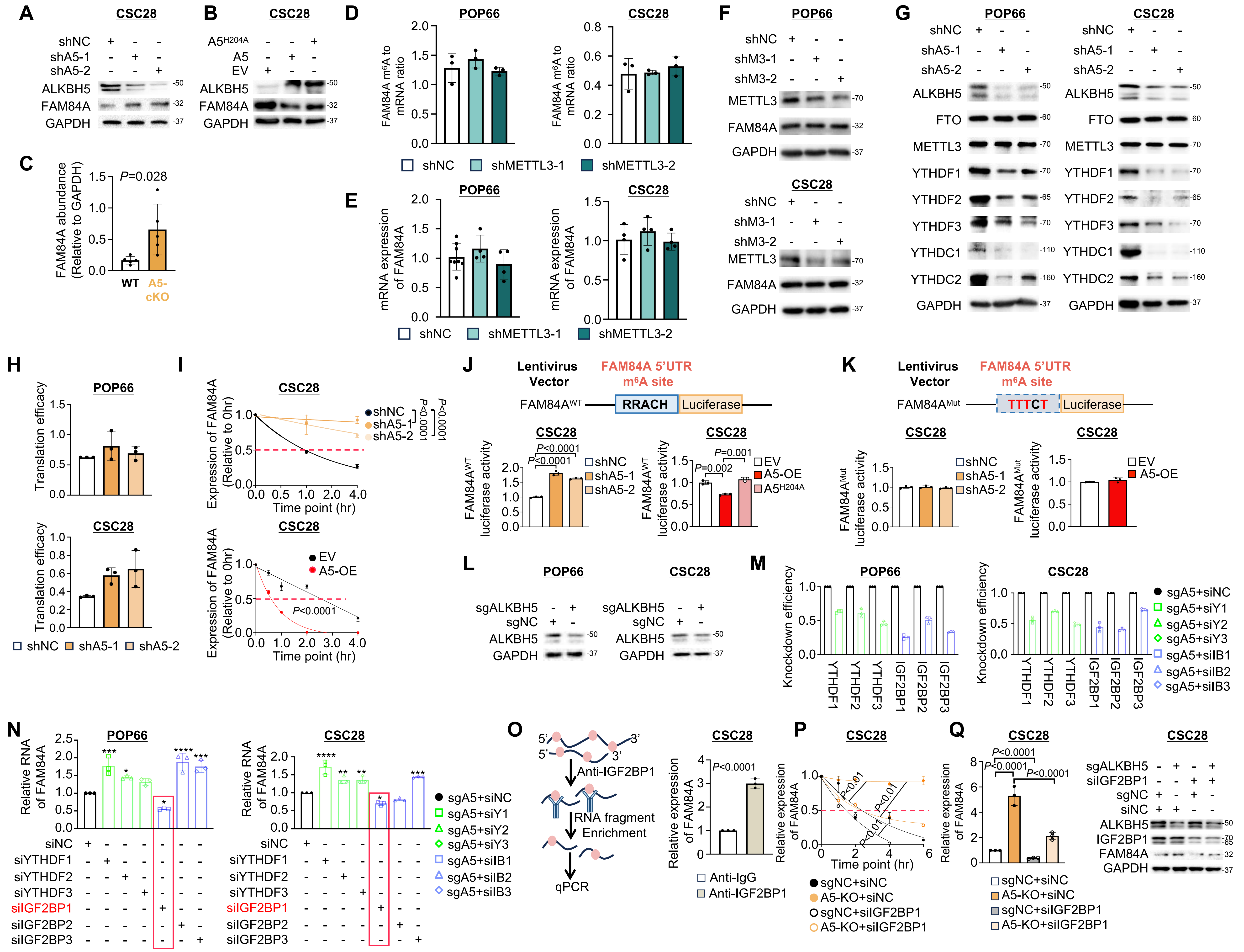

Figure S6. ALKBH5 modulates FAM84A mRNA stability via IGF2BP1.

(A-B) FAM84A expression as determined by western blot in CSC28. **(C)** Densitometry quantification of FAM84A in CRC tissues of WT versus ALKBH5-cKO mice (n=5, each dot represents an independent mouse). **(D)** m^{6}A modification on FAM84A in shNC cells compared with shMETTL3 cells as determined by MeRIP-qPCR (n=3, each dot represents an independent sample). **(E)** mRNA abundance of FAM84A in shNC cells compared with shMETTL3 cells as determined by RT-qPCR (For POP66, n=8 in shNC, n=4 in shM3-1, n=4 in shM3-2; For CSC28, n=4, each dot represents an independent sample). **(F)** Protein abundance of FAM84A in shNC cells compared with shMETTL3 cells as determined by western blot. **(G)** Protein abundance of m^{6}A regulators in shNC cells compared with shALKBH5 cells as determined by western blot. **(H)** Translation efficiency of FAM84A with ALKBH5 knockdown in POP66 and CSC28 (n=3, each dot represents an independent sample). **(I)** FAM84A mRNA stability as determined by qPCR after ALKBH5 knockdown in CSC28. RNA decay rate was normalized to expression at 0 hr (upper panel). FAM84A mRNA stability as determined by qPCR after ALKBH5 overexpression in CSC28. RNA decay rate was normalized to expression at 0 hr (lower panel) (n=3, each dot represents an independent sample). **(J)** Plasmid design for the luciferase reporter assay with FAM84A^{WT} 5'UTR (upper panel), luciferase activity of FAM84A^{WT} reporter with ALKBH5 knockdown and FAM84A^{WT} reporter with the overexpression of WT ALKBH5 (A5-OE) or mutant ALKBH5 (A5^{H204A}) in CSC28 (lower panel) (n=3, each dot represents an independent sample). **(K)** Plasmid design for luciferase reporter assay with mutated 5'UTR (FAM84A^{Mut}) sequences added to the 5' end of luciferase gene (upper panel), luciferase activity of FAM84A^{Mut} reporter with ALKBH5 knockdown and FAM84A^{Mut} reporter with the overexpression of WT ALKBH5 (A5-OE) in POP66 (lower panel) (n=3, each dot represents an independent sample). **(L)** Validation of ALKBH5 knockout efficacy by western blot. **(M)** Validation of m^{6}A reader knockdown efficacy as determined by qPCR (n=3, each dot represents an independent sample). **(N)** FAM84A mRNA expression as determined by qPCR (n=3, each dot represents an independent sample). **(O)** Schematic diagram for RIP-qPCR assay (left panel). Binding between IGF2BP1 protein and FAM84A mRNA was determined by RIP-qPCR in CSC28 (right panel) (n=3, each dot represents an independent sample). **(P)** Effect of IGF2BP1 knockdown on the stability of FAM84A mRNA in CSC28 with or without ALKBH5 knockout (n=4, each dot represents an independent sample). **(Q)** Effect of IGF2BP1 knockdown on FAM84A mRNA (n=3, each dot represents an independent sample) (left panel) and protein (right panel) in CSC28 with or without ALKBH5 knockout.

Results are presented as mean ± S.D. Each spot represents one subject. Statistical significance was determined by student's t test.

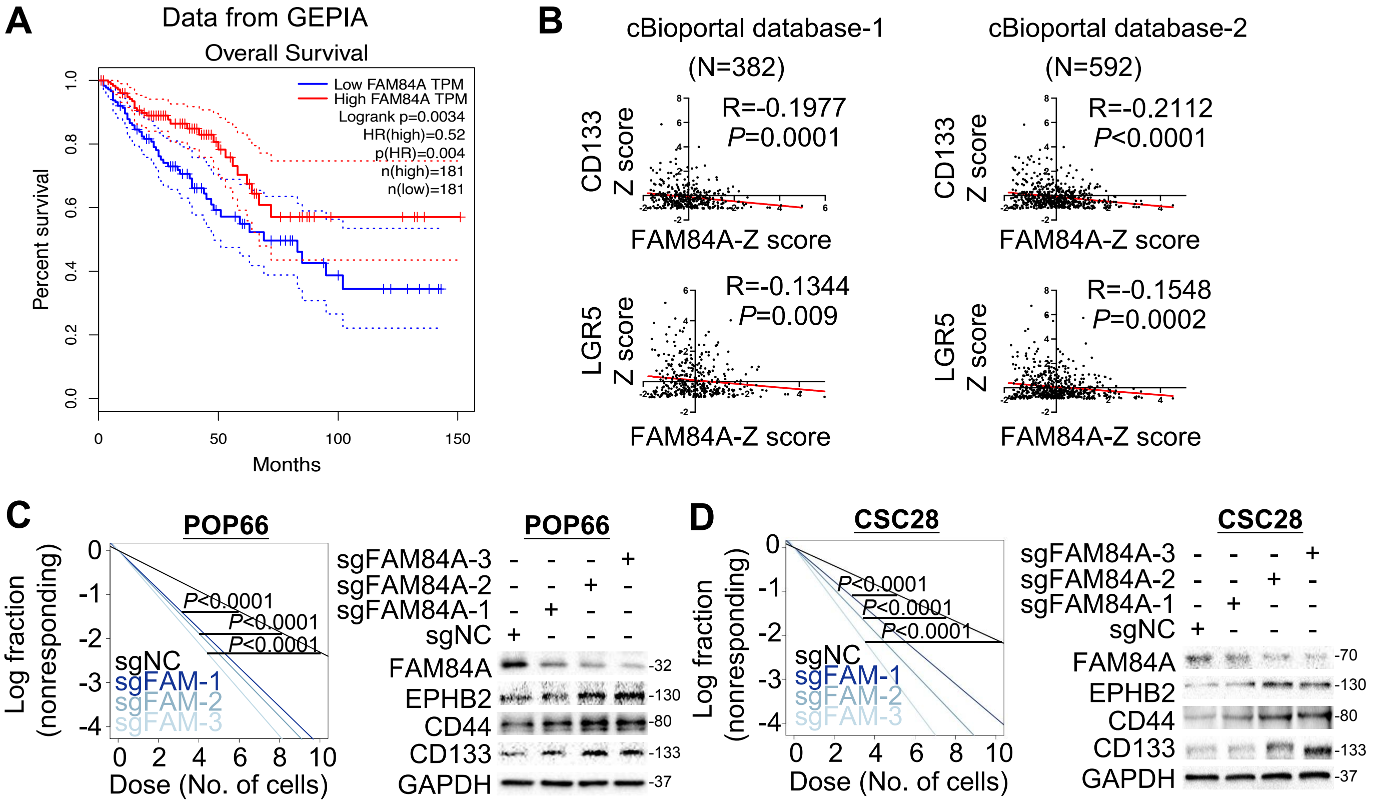

Figure S7. FAM84A is a tumor suppressive gene in colorectal CSCs.
(A) Survival analysis of FAM84A in GEPIA website. **(B)** Correlation between FAM84A and stemness markers (CD133, LGR5) in cBioportal database. **(C-D)** FAM84A knockout promoted stemness properties, as determined by *in vitro* LDA and stemness markers (CD133, CD44 and EPHB2) in POP66 and CSC28.

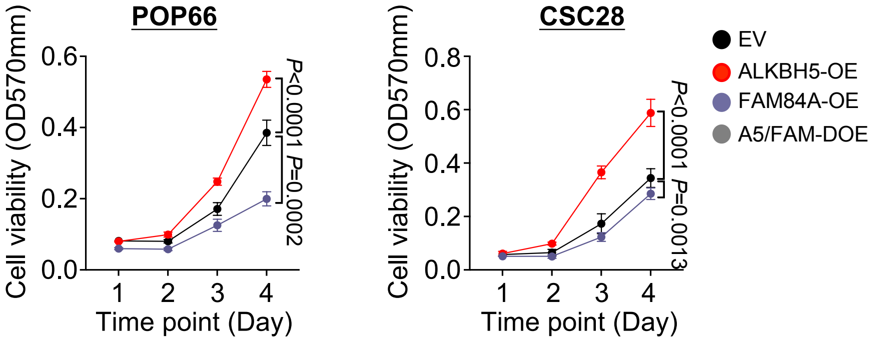

Figure S8. FAM84A overexpression abolishes ALKBH5-induced proliferation in colorectal CSCs. Effect of FAM84A overexpression on the proliferation of POP66- and CSC28-overexpressing ALKBH5, as determined by MTT assay (n=4, each dot represents an independent sample).

Results are presented as mean ± S.D. Each spot represents one subject. Statistical significance was determined by student's t test or two-way ANOVA where appropriate.

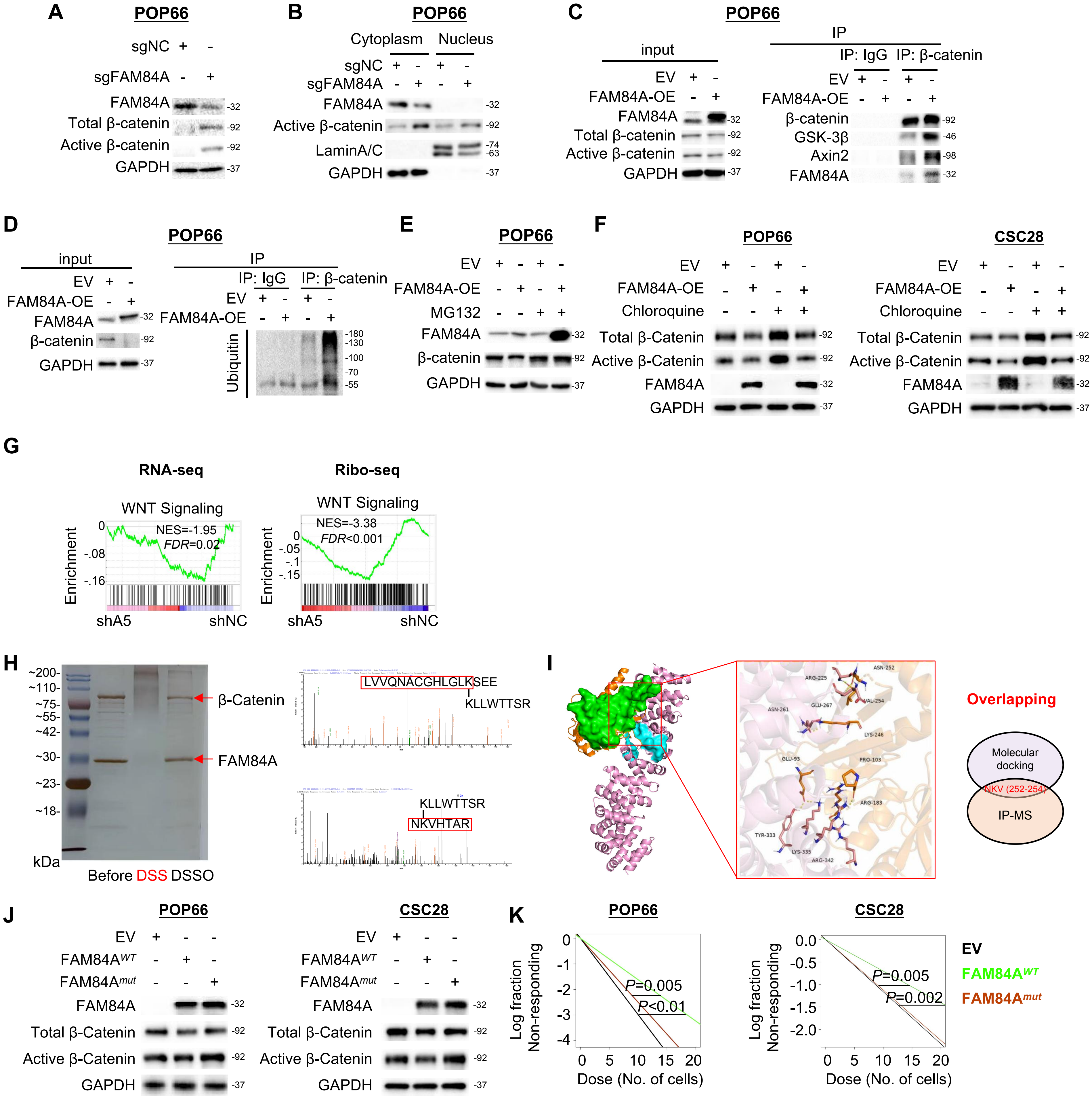

Figure S9. FAM84A axis regulates β-catenin degradation in colorectal CSCs.
(A) Abundance of total β-catenin and active β-catenin as determined by western blot in POP66. **(B)** Abundance of active β-catenin as determined by western blot analysis of cytoplasm and nuclear protein fractions from POP66. **(C)** Expression of total β-catenin and active β-catenin as determined by western blot and their interaction with GSK-3β and Axin-2 as revealed by co-immunoprecipitation assay in POP66. **(D)** β-catenin expression and ubiquitination in POP66. **(E)** Expression of total β-catenin as determined by western blot with or without MG132 treatment in POP66. **(F)** Expression of total β-catenin and active β-catenin as determined by western blot with or without Chloroquine treatment in POP66 and CSC28. **(G)** WNT Signaling enriched based on DEGs in ALKBH5-knockdown (shA5) vs. control CSC28 cells by RNA-seq (left panel) and Ribo-seq (right panel) with p-value. **(H)** Cross-linking between FAM84A and β-catenin as determined by silver staining (left panel) with following result of IP-MS (right panel). **(I)** Molecular docking of interplay between FAM84A and β-catenin. **(J)** Expression of total β-catenin and active β-catenin as determined by western blot among EV, FAM84A^{WT} and FAM84A^{mut} cells. **(K)** Self-renewal ability as determined by *in vitro* LDA among EV cells, FAM84A^{WT} cells and FAM84A^{mut} cells.
Results are presented as mean ± S.D. Each spot represents one subject. Statistical significance was determined by student's *t* test or one-way ANOVA where appropriate.

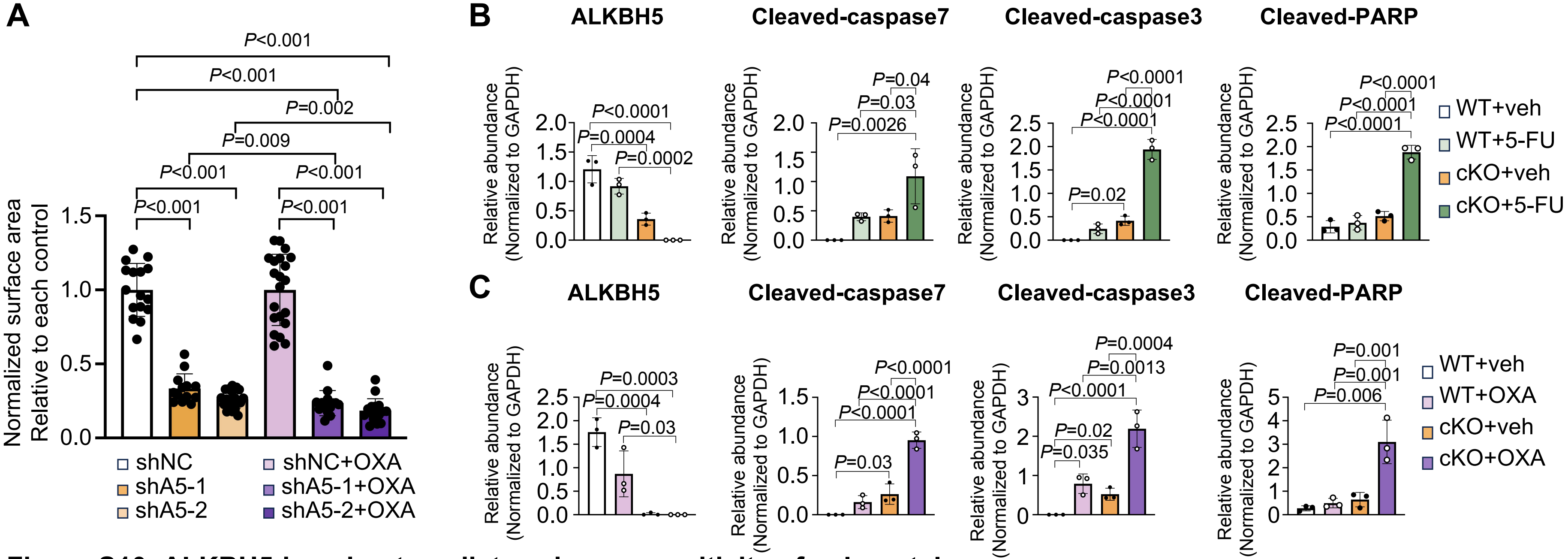

Figure S10. ALKBH5-knockout mediates chemo-sensitivity of colorectal cancer.

(A) Normalized surface area of every group (Relative to shNC-OXA$^{-/+}$ group) (n=16 for shNC, n=13 for shA5-1, n=21 for shA5-2, n=21 for shNC+OXA, n=15 for shA5-1+OXA, n=16 for shA5-2+OXA, each dot represents an independent captured view) **(B)** Quantitative analysis of apoptosis markers in cKO mice or WT mice after 5-FU treatment) (n=3, each dot represents an independent mouse). **(C)** Quantitative analysis of apoptosis markers abundance in cKO mice or WT mice after Oxaliplatin treatment (n=3, each dot represents an independent mouse).
Results are presented as mean ± S.D. Each spot represents one subject. Statistical significance was determined by student's *t* test or one-way ANOVA where appropriate.

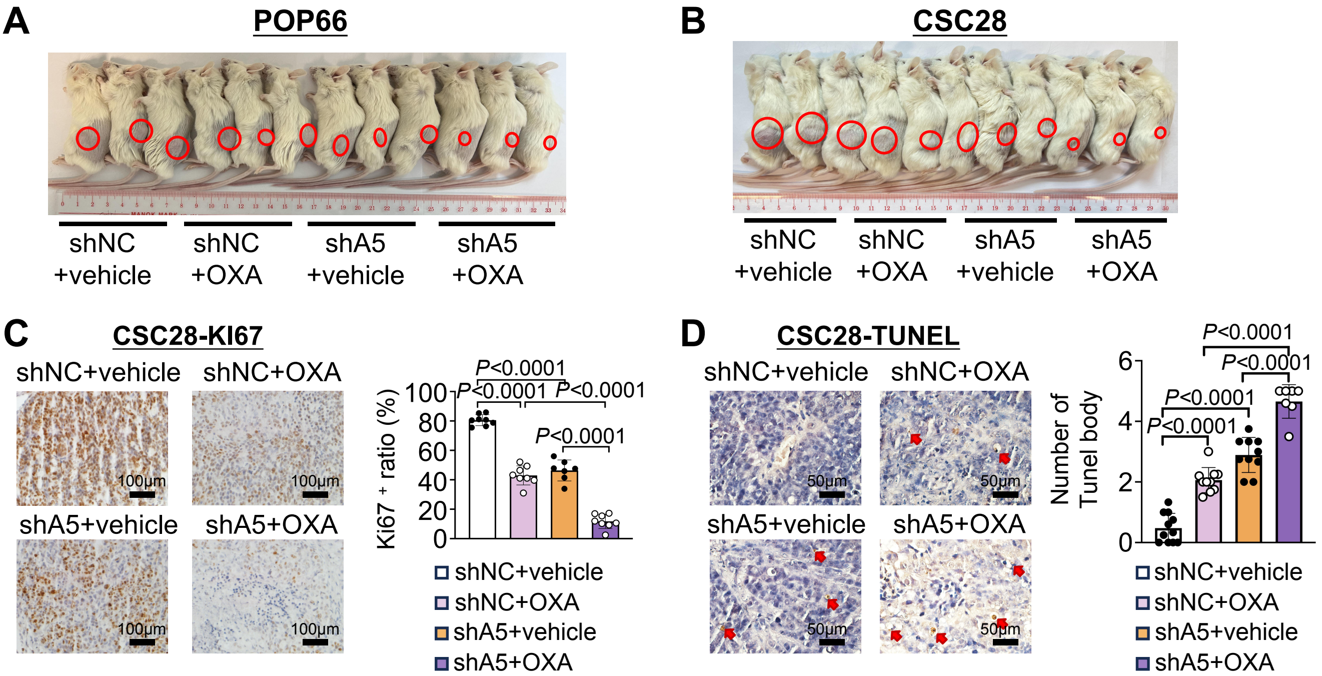

Figure S11. ALKBH5 knockdown increased Oxaliplatin efficacy in colorectal CSCs.

(A) Representative image of mice in POP66 xenograft model with or without ALKBH5 knockdown after Oxaliplatin treatment. **(B)** Representative image of mice in CSC28 xenograft model with or without ALKBH5 knockdown after Oxaliplatin treatment. **(C)** Ki67^{+} (anti-Ki67 antibody) cells as determined by IHC in CSC28 (shNC+ vehicle: n=8; shNC+ OXA: n=8; shA5+ vehicle: n=7; shA5+ OXA: n=8; each dot represents an independent captured view). **(D)** Apoptosis as determined by TUNEL staining in CSC28 xenograft (shNC+ vehicle: n=11; shNC+ OXA: n=10; shA5+ vehicle: n=10; shA5+ OXA: n=6; each dot represents an independent captured view).

Results are presented as mean ± S.D. Each spot represents one subject. Statistical significance was determined by one-way ANOVA.

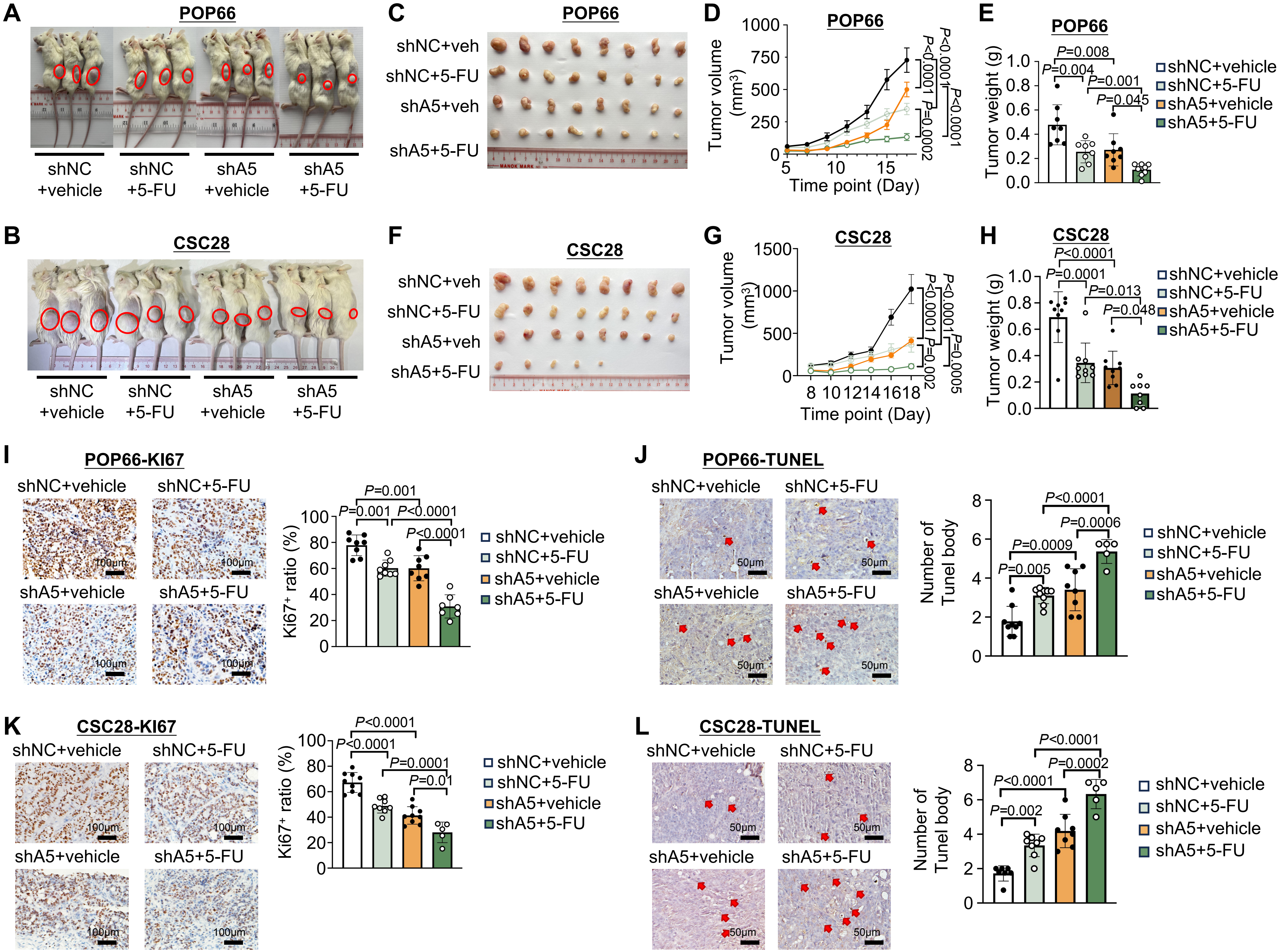

Figure S12. ALKBH5 knockdown increased 5-FU-efficacy in colorectal CSCs.

(A) Representative image of POP66 xenografts with or without ALKBH5 knockdown after 5-FU treatment. **(B)** Representative image of CSC28 xenografts with or without ALKBH5 knockdown after 5-FU treatment. **(C)** Image of xenografts, **(D)** Tumor growth, and **(E)** tumor weight of POP66 with or without ALKBH5 knockdown after 5-FU treatment (n=8; each dot represents an independent mouse). **(F)** Image of xenografts, **(G)** tumor growth, and **(H)** tumor weight of CSC28 with or without ALKBH5 knockdown after 5-FU treatment (shNC+ vehicle: n=9; shNC+ 5-FU: n=9; shA5+ vehicle: n=9; shA5+ 5-FU: n=8; each dot represents an independent mouse). **(I)** Ki67⁺ cells (shNC+ vehicle: n=8; shNC+ 5-FU: n=8; shA5+ vehicle: n=8; shA5+ 5-FU: n=7; each dot represents an independent patured view) and **(J)** apoptosis proportion as determined by TUNEL staining in POP66 xenografts (shNC+ vehicle: n=9; shNC+ 5-FU: n=9; shA5+ vehicle: n=8; shA5+ 5-FU: n=5; each dot represents an independent captured view). **(K)** Ki67⁺ cells (shNC+ vehicle: n=9; shNC+ 5-FU: n=8; shA5+ vehicle: n=9; shA5+ 5-FU: n=5; each dot represents an independent captured view) and **(L)** apoptosis proportion as determined by TUNEL staining (shNC+ vehicle: n=7; shNC+ 5-FU: n=8; shA5+ vehicle: n=8; shA5+ 5-FU: n=8; each dot represents an independent capture view) in CSC28 xenografts. Results are presented as mean ± S.D or mean ± SEM. Each spot represents one subject. Statistical significance was determined by one-way ANOVA or two-way ANOVA where appropriate.

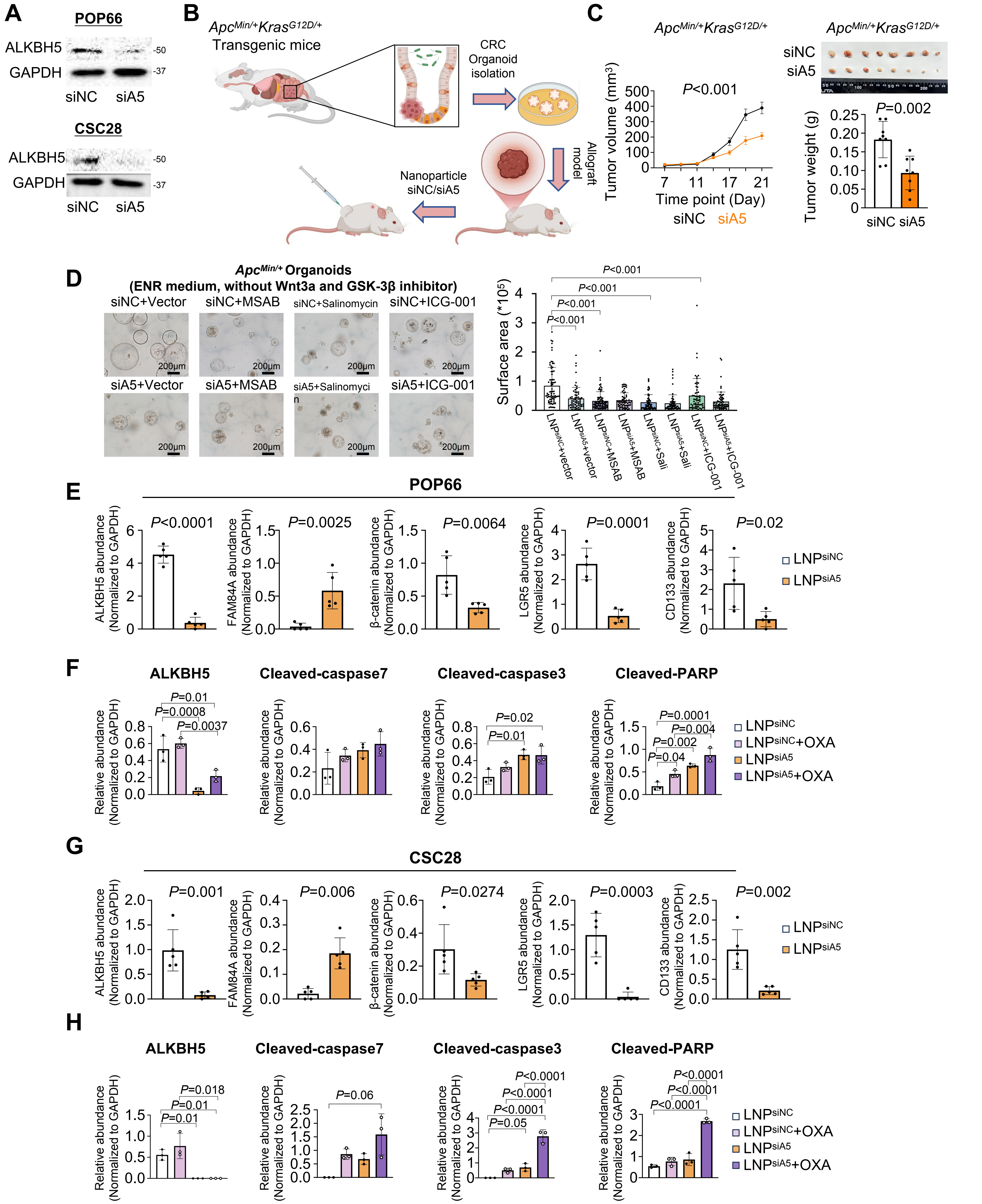

Figure S13. Efficacy of nanoparticle-encapsulated siALKBH5.

(A) Knockdown of ALKBH5 was validated by western blot in POP66 and CSC28. **(B)** Workflow of xenograft model based on *APC*$^{Min/+}$*KRAS*$^{G12D/+}$ (AK) CRC organoids (ENR culture medium) (The diagram is created Created in BioRender. Chou, H. (2025) https://BioRender.com/cg01pkr). **(C)** Tumor growth curve of AK organoids treated with nano-siNC or nano-siA5 (left panel). Tumor weight of AK organoids treated with nano-siNC or nano-siA5 (right panel) (n=8; each dot represents an independent mouse). **(D)** Self-renewal ability as determined by surface area of *APC*$^{Min/+}$ tumor organoids (ENR culture medium) with or without wnt inhibitor treatment in nano-siNC or nano-siA5 organoids (siNC+ vehicle: n=74; siA5+vehicle: n=63; siNC+ MSAB: n=79; siA5+ MSAB: n=61; siNC+ Sali: n=70; siA5+ Sali: n=69; siNC+ ICG: n=59; siA5+ ICG: n=68; each dot represents an independent organoid). **(E)** Quantitative analysis of ALKBH5, FAM84A, Active β-catenin, LGR5, CD133 (n=5, each dot represents an independent mouse) and **(F)** apoptosis markers abundance (n=3, each dot represents an independent mouse) (normalized to GAPDH) in tumor tissue from POP66 xenografts. **(G)** Quantitative analysis of ALKBH5, FAM84A, Active β-catenin, LGR5, CD133 (n=5, each dot represents an independent mouse) and **(H)** apoptosis markers abundance (n=3, each dot represents an independent mouse) (normalized to GAPDH) in tumor tissue from CSC28 xenografts.

Results are presented as mean ± S.D or mean ± SEM. Each spot represents one subject. Statistical significance was determined by one-way ANOVA or two-way ANOVA where appropriate.

Legends for Supplementary Data:

Legend for Supplementary Data 1:
Gene list of AmpliSeq for Illmina Cancer HotSpot Panel.

Legend for Supplementary Data 2:
Enriched m^6A motifs with p-values in MeRIP seq.

Supplementary Table 1 siRNA sequencing

	sense (5'-3')	Antisense (5'-3')
ALKBH5	CUGCGCAACAAGUACUUCUTT	AGAAGUACUUGUUGCGCAGTT
YTHDF1-1	CCUCCACCCAUAAAGCAUATT	UAUGCUUUAUGGGUGGAGGTT
YTHDF1-2	GCUCCAUUAAGUACUCCAUTT	AUGGAGUACUUAAUGGAGCTT
YTHDF2-1	GCUCUGGAUAUAGUAGCAATT	UUGCUACUAUAUCCAGAGCTT
YTHDF2-2	GCGGGUCCAUUACUAGUAATT	UUACUAGUAAUGGACCCGCTT
YTHDF3-1	GGGACAAUCAACACAAAGUTT	ACUUUGUGUUGAUUGUCCCTT
YTHDF3-2	GACUAGCAUUGCAACCAAUTT	AUUGGUUGCAAUGCUAGUCTT
IGF2BP1-1	CCCAGUAUGUGGGUGCCAUTT	AUGGCACCCACAUACUGGGTT
IGF2BP1-2	CCAAAGUUCGUAUGGUUAUTT	AUAACCAUACGAACUUUGGTT
IGF2BP2-1	GUCAACGUCACAUAUGCAATT	AUGCUGCUGCUUAACCUGGTT
IGF2BP2-2	GAGGGCUUGACCAUAAAGATT	AUUCCACUUUACCCGAGAGTT
IGF2BP3-1	GGAUUCGGAAACUUCAGAUTT	AUCUGAAGUUUCCGAAUCCTT
IGF2BP3-2	GCUGCUGAGAAGUCGAUUATT	UAAUCGACUUCUCAGCAGCTT

Supplementary Table 2 Primer sequences

**Real-time PCR**	
ALKBH5-F	ATCCTCAGGAAGACAAGATTAG
ALKBH5-R	TTCTCTTCCTTGTCCATCTC
GAPDH-F	AATGGGCAGCCGTTAGGAAA
GAPDH-R	GCCCAATACGACCAAATCAGAG
β -actin-F	CATTGCTGACAGGATGCAGAAGG
β -actin-R	TGCTGGAAGGTGGACAGTGAGG
YTHDF1-F	TGGACACCCAGAGAACAAAAGG
YTHDF1-R	CCAATGGACGGCGGGTAATA
YTHDF2-F	CTGTTGGTAGCGGGTCCATT
YTHDF2-R	GCCCAAGATGCTGGTTTTGG
YTHFD3-F	TGTGGGAATTGGGGGTTCTG
YTHDF3-R	GGAGCCTTTACCACTGACCC
IGF2BP1-F	ATCGGCAACCTCAACGAGAG
IGF2BP1-R	GTTTCGATGGCCTTCATCGC
IGF2BP2-F	CTACGCCTTCGTGGACTACC
IGF2BP2-R	TGTTGACTTGTTCCACATTCTCC
IGF2BP3-F	ACTGCACGGGAAACCCATAG
IGF2BP3-R	TCCCACTGTAAATGAGGCGG
**MeRIP-qPCR**	
FAM84A-F	ACGATTCGGCTCGGGGTA
FAM84A-R	GCTTCTCCCTACCCGTGAT

Supplementary Table 3 CRISPR/Cas9 sgRNA

ALKBH5-sgRNA-F	CACCGTCTCGTCCACGTCGCCCGG
ALKBH5-sgRNA-R	AAACCCGGGCGACGTGGACGAGAC
FAM84A-sgRNA-1	CACCGATTCACCACCCGGCCCACGTGTTT
FAM84A-sgRNA-2	CACCGACCGCGTAGACGCTTAGGTGTTT
FAM84A-sgRNA-3	CACCGCTACAGCGAGTTGCCCACAGGTTT

Supplementary Table 4 gRNA for dCas13b-ALKBH5 system

FAM84A-gRNA-1	GATTTCCCTGCTCCCGAAACCCC
FAM84A-gRNA-2	AAACTCCTTTAAAGTCTCCTGGT
FAM84A-gRNA-3	CGTGGGCCGGGTGGTGAATAGCTGGTAC

Supplementary Table 5 Mutation profile of PDOs

**PDO-816**		
Gene	Variant Type	Amino Acids Changed
NRAS	missense _variant	G13V
APC	stop _ gained	NM_000038.5:c.4099C>T
**PDO-828**		
Gene	Variant Type	Amino Acids Changed
APC	stop _ gained	NM_000038.5:c.4495G>T
BRAF	missense _variant	V600E
PTEN	missense _variant	C105W
TP53	missense _variant	R248W

Supplementary Table 6 Antibody list

REAGENT	SOURCE	IDENTIFIER
**Antibodies**		
GAPDH	Cell Signaling Technology	Cat # 5174
ALKBH5	Sigma-Aldrich	Cat # HPA001796
LGR5	Origene	Cat # TA503316
EGFP	Abcam	Cat # Ab6556
CD133	Cell Signaling Technology	Cat # 5860
Anti-mouse Cd133	Abcam	Cat # Ab271092
CD44	Cell Signaling Technology	Cat # 3570
FAM84A	Santa Cruz Biotechnology	Cat # sc-101207
Total β-Catenin	Cell Signaling Technology	Cat # 8480
Active β-Catenin	Cell Signaling Technology	Cat # 19807
AXIN2	Abcam	Cat # Ab109307
GSK3β	Cell Signaling Technology	Cat # 12456
KI67	Cell Signaling Technology	Cat # 9129
N6-methyladenosine	Abcam	Cat # Ab208577
IGF2BP1	Proteintech	Cat # 22803-1-AP
LAMIN A/C	Cell Signaling Technology	Cat # 4777
m^6A	Abcam	Cat # ab208577
Ki67-FITC	Biolegend	Cat # 151212
Cd133-PE	Biolegend	Cat # 141204
GFP-PE	Biolegend	Cat # 338003

Supplementary Table 7 chemical list

Chemicals		
HEPES	Gibco	Cat # 15630080
Penicillin-Streptomycin antibiotics	Gibco	Cat # 15140122
Primocin	Invivogen	Cat # ant-pm-1
Glutamax	Gibco	Cat # 35050061
B-27 Supplement	Gibco	Cat # 17504044
N-2 Supplement	Gibco	Cat # 17502048
N-Acetylcysteine	Sigma-Aldrich	Cat # A9165-5G
A8301	Tocris	Cat # 2938
SB202190	Stem Cell Technology	Cat # 72632
CHIR99021	Tocris	Cat # 4423/10
Nicotinamide	Sigma-Aldrich	Cat # N0636
Y27632	Stem cell technology	Cat # 72305
FGF10	Preprotech	Cat # 100-26
mEGF	Gibco	Cat # PMG8041
Azoxymethane (AOM)	Sigma-Aldrich	Cat # A5486
Dextran Sulfate Sodium (DSS)	MP Biomedicals	Cat # MFCD00081551
5-FU	MedChemExpress	Cat#: HY-90006
Oxaliplatin	MedChemExpress	Cat#: HY-17317
MG132	MedChenExpress	Cat#: HY-13259
Chloroquine	MedChenExpress	Cat#: HY-17589A
MSAB	MedChenExpress	Cat#: HY-120697
Salinomycin	MedChenExpress	Cat#: HY-15597
ICG-001	MedChenExpress	Cat#: HY-14428
