# Supplementary material for: Targeting of the m6A eraser ALKBH5 suppresses stemness and chemoresistance of colorectal cancer
Source: Nat Commun. 2025 Dec 13;17:803. doi: 10.1038/s41467-025-67502-0 (PMC12824147; doi:10.1038/s41467-025-67502-0)
Supplement: Supplementary file 2 — Description of Additional Supplementary Files [file 41467_2025_67502_MOESM2_ESM.pdf]

Description of Additional Supplementary Files

Supplementary Data 1:
Gene list of AmpliSeq for Illmina Cancer HotSpot Panel.

Supplementary Data 2:
Enriched m^6A motifs with *p*-values in MeRIP seq.
