# Supplementary material for: Targeting of the m6A eraser ALKBH5 suppresses stemness and chemoresistance of colorectal cancer
Source: Nat Commun. 2025 Dec 13;17:803. doi: 10.1038/s41467-025-67502-0 (PMC12824147; doi:10.1038/s41467-025-67502-0)
Supplement: Supplementary file 5 — Reporting Summary [file 41467_2025_67502_MOESM5_ESM.pdf]

nature portfolio

Corresponding author(s): DAPR-NCOMMS-24-52715B

Last updated by author(s): Oct 3, 2025

Reporting Summary

Nature Portfolio wishes to improve the reproducibility of the work that we publish. This form provides structure for consistency and transparency in reporting. For further information on Nature Portfolio policies, see our Editorial Policies and the Editorial Policy Checklist.

Statistics

For all statistical analyses, confirm that the following items are present in the figure legend, table legend, main text, or Methods section.

n/a	Confirmed	
☐	☒	The exact sample size (*n*) for each experimental group/condition, given as a discrete number and unit of measurement
☐	☒	A statement on whether measurements were taken from distinct samples or whether the same sample was measured repeatedly
☐	☒	The statistical test(s) used AND whether they are one- or two-sided<br>*Only common tests should be described solely by name; describe more complex techniques in the Methods section.*
☒	☐	A description of all covariates tested
☐	☒	A description of any assumptions or corrections, such as tests of normality and adjustment for multiple comparisons
☐	☒	A full description of the statistical parameters including central tendency (e.g. means) or other basic estimates (e.g. regression coefficient) AND variation (e.g. standard deviation) or associated estimates of uncertainty (e.g. confidence intervals)
☐	☒	For null hypothesis testing, the test statistic (e.g. F, t, r) with confidence intervals, effect sizes, degrees of freedom and P value noted<br>*Give P values as exact values whenever suitable.*
☒	☐	For Bayesian analysis, information on the choice of priors and Markov chain Monte Carlo settings
☒	☐	For hierarchical and complex designs, identification of the appropriate level for tests and full reporting of outcomes
☒	☐	Estimates of effect sizes (e.g. Cohen's *d*, Pearson's *r*), indicating how they were calculated

Our web collection on statistics for biologists contains articles on many of the points above.

Software and code

Policy information about availability of computer code

Data collection	Image Lab software v6.1.0 (Bio-rad), Quantstudio Real Time PCR software v1.7.2 (Applied Biosystems), Leica Las X v5.02 (Leica).
Data analysis	FlowJo v10 (BD Biosciences), Prism 9.0 (GraphPad Software, Inc).

For manuscripts utilizing custom algorithms or software that are central to the research but not yet described in published literature, software must be made available to editors and reviewers. We strongly encourage code deposition in a community repository (e.g. GitHub). See the Nature Portfolio guidelines for submitting code & software for further information.

Data

Policy information about availability of data

All manuscripts must include a data availability statement. This statement should provide the following information, where applicable:

- Accession codes, unique identifiers, or web links for publicly available datasets
- A description of any restrictions on data availability
- For clinical datasets or third party data, please ensure that the statement adheres to our policy

We have uploaded all the sequencing data to SRA with reviewer link provided.

Research involving human participants, their data, or biological material

Policy information about studies with human participants or human data. See also policy information about sex, gender (identity/presentation), and sexual orientation and race, ethnicity and racism.

Reporting on sex and gender	The informed consent for the study specimens were obtained from all subjects. This study was approved by Clinical Research Ethics Committee of the Chinese University of Hong Kong. All patients were treatment naive CRC patients. The age and gender of the patients have been documented.
Reporting on race, ethnicity, or other socially relevant groupings	N/A
Population characteristics	Tumor tissues were collected from patients with pathologically confirmed CRC undergoing surgery at the Prince of Wales Hospital, the Chinese University of Hong Kong.
Recruitment	Patients were enrolled in by their oncologist at Prince of Wales Hospital, the Chinese University of Hong Kong, and Peking University Cancer Hospital. All patients provided written informed consent for obtaining the study specimens.
Ethics oversight	The study protocols were approved by the Clinical Research Ethics Committee of Prince of Wales Hospital, the Chinese University of Hong Kong, and Peking University Cancer Hospital. All patients provided written informed consent for obtaining the study specimens. This study was performed in accordance with the declaration of Helsinki of the World Medical Association.

Note that full information on the approval of the study protocol must also be provided in the manuscript.

Field-specific reporting

Please select the one below that is the best fit for your research. If you are not sure, read the appropriate sections before making your selection.

- [x] Life sciences
- [ ] Behavioural & social sciences
- [ ] Ecological, evolutionary & environmental sciences

For a reference copy of the document with all sections, see nature.com/documents/nr-reporting-summary-flat.pdf

Life sciences study design

All studies must disclose on these points even when the disclosure is negative.

Sample size	Sample size for in vivo and in vitro work was decided based on experience from similar experiments in our laboratory (Jianning Zhai et al. Gastroenterology, PMID: 37169182; Huarong Chen et al. Gastroenterology, PMID: 35700773; Huarong Chen et al. Gastroenterology, PMID: 33217448; Qiming Zhou et al. Nature Communications, PMID: 37542037)
Data exclusions	No exclusion of data was performed.
Replication	All in vitro work was repeated in at least 2 independent experiments. Details of experimental replicates are given in the figure legends. All reported attempts at replication were successful.
Randomization	All samples and animals were analyzed and allocated randomly.
Blinding	Pathological evaluation was performed in a blinded manner by experienced pathologists.

Reporting for specific materials, systems and methods

We require information from authors about some types of materials, experimental systems and methods used in many studies. Here, indicate whether each material, system or method listed is relevant to your study. If you are not sure if a list item applies to your research, read the appropriate section before selecting a response.

Materials & experimental systems

n/a	Involved in the study	
[ ]	[x]	Antibodies
[ ]	[x]	Eukaryotic cell lines
[x]	[ ]	Palaeontology and archaeology
[ ]	[x]	Animals and other organisms
[x]	[ ]	Clinical data
[x]	[ ]	Dual use research of concern
[x]	[ ]	Plants

Methods

n/a	Involved in the study	
[x]	[ ]	ChIP-seq
[ ]	[x]	Flow cytometry
[x]	[ ]	MRI-based neuroimaging

Antibodies

Antibodies used	All antibodies used in this study have been listed in Supplementary Methods_Reagents Table.
Validation	All antibodies used in this study have been validated according to protocol on the antibody website.

Eukaryotic cell lines

Policy information about cell lines and Sex and Gender in Research

Cell line source(s)	Human colon CSC-enriched spheroid models (POP66 and CSC28) and colon cancer patient-derived organoids (PDOs) (816 and 828) were kindly provided by Dr. Catherine Adell O'Brein in Princess Margaret Cancer Center, University of Toronto. POP66 and CSC28 were isolated from liver metastases species from colon adenocarcinoma and cultured in 3D suspension state in previously established serum-free, growth factor-enriched medium which suitable for CSCs. PDO-828 was isolated from a primary colon tumor and PDO-816 was isolated from lung metastases of colon cancer.
Authentication	Human colon CSC-enriched spheroid models (POP66 and CSC28) and colon cancer patient-derived organoids (PDOs) (816 and 828) were kindly provided by Dr. Catherine Adell O'Brein in Princess Margaret Cancer Center, University of Toronto.
Mycoplasma contamination	We have confirmed that all cell lines tested negative for mycoplasma contamination.
Commonly misidentified lines (See ICLAC register)	N/A

Animals and other research organisms

Policy information about studies involving animals; ARRIVE guidelines recommended for reporting animal research, and Sex and Gender in Research

Laboratory animals	3-4 week-old male NOD scid gamma (NSG) mice were obtained from the Chinese University of Hong Kong mouse repository. Conditional Alkbh5 knockin mice (Rosa26lsl-Alkbh5) were generated by Shanghai Model Organisms Company, which were crossed to LGR5-CreERT2 mice to achieve colon stem cell-specific Alkbh5 knockin mice (Rosa26lsl-Alkbh5LGR5-CreERT2). To generate the Alkbh5-/- mice, single-guide RNAs (sgRNAs) targeting exon 1 of Alkbh5 were used, which resulted in Alkbh5 knockout (Alkbh5flox/flox). Mice were again crossed to LGR5-CreERT2 mice to achieve colon stem cell-specific Alkbh5 knockout mice (Alkbh5flox/flox LGR5-CreERT2). The mice were maintained on a C57BL/6 background. All animal studies are approved by the Animal Experimentation Ethics Committee, CUHK.<br>All mice were maintained under specific pathogen-free conditions at the animal facility of CUHK. Mice were maintained in 12 hour light/dark cycle, and the housing temperature and humidity were 23 degrees and 45%, respectively.
Wild animals	N/A
Reporting on sex	All mice used in the experiments were male.
Field-collected samples	N/A
Ethics oversight	The study protocols were approved by the Clinical Research Ethics Committee of Prince of Wales Hospital, the Chinese University of Hong Kong.

Note that full information on the approval of the study protocol must also be provided in the manuscript.

Plants

Seed stocks	*Report on the source of all seed stocks or other plant material used. If applicable, state the seed stock centre and catalogue number. If plant specimens were collected from the field, describe the collection location, date and sampling procedures.*
Novel plant genotypes	*Describe the methods by which all novel plant genotypes were produced. This includes those generated by transgenic approaches, gene editing, chemical/radiation-based mutagenesis and hybridization. For transgenic lines, describe the transformation method, the number of independent lines analyzed and the generation upon which experiments were performed. For gene-edited lines, describe the editor used, the endogenous sequence targeted for editing, the targeting guide RNA sequence (if applicable) and how the editor was applied.*
Authentication	*Describe any authentication procedures for each seed stock used or novel genotype generated. Describe any experiments used to assess the effect of a mutation and, where applicable, how potential secondary effects (e.g. second site T-DNA insertions, mosiacism, off-target gene editing) were examined.*

Flow Cytometry

Plots

Confirm that:

- [x] The axis labels state the marker and fluorochrome used (e.g. CD4-FITC).
- [x] The axis scales are clearly visible. Include numbers along axes only for bottom left plot of group (a 'group' is an analysis of identical markers).
- [x] All plots are contour plots with outliers or pseudocolor plots.
- [x] A numerical value for number of cells or percentage (with statistics) is provided.

Methodology

Sample preparation	3D suspension cancer stem cell spheres were dissected and digested by TE and stained by PI and FITC. Samples was filtered by 40 um cell strainer to obtain single cell.
Instrument	BD FACS Celesta
Software	FlowJo v10
Cell population abundance	Abundance and purity of cell population determined by FlowJo v10 software with identical gating strategy across each set of samples.
Gating strategy	FSC-A/SSC-A was used for gating cancer stem cells. PI and FITC were used for gating apoptosis cells.

- [x] Tick this box to confirm that a figure exemplifying the gating strategy is provided in the Supplementary Information.
