# Supplementary material for: Targeting of the m6A eraser ALKBH5 suppresses stemness and chemoresistance of colorectal cancer
Source: Nat Commun. 2025 Dec 13;17:803. doi: 10.1038/s41467-025-67502-0 (PMC12824147; doi:10.1038/s41467-025-67502-0)
Supplement: Supplementary file 7 — Source Data [file 41467_2025_67502_MOESM7_ESM.zip › Source Data/Supplementary inforamtion_Gating strategy.pdf]

Gating strategy for **Figure 1R**

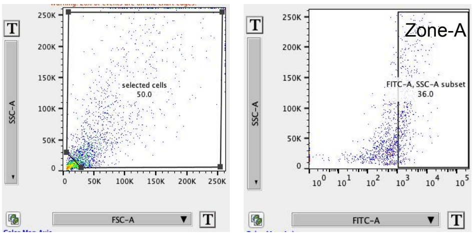

Gating strategy for **Figure 1S**

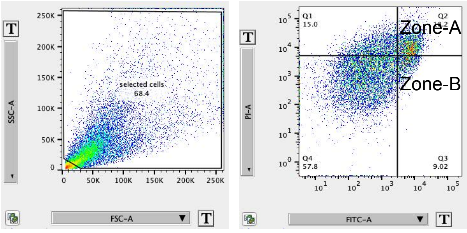

Gating strategy for **Figure 1T**

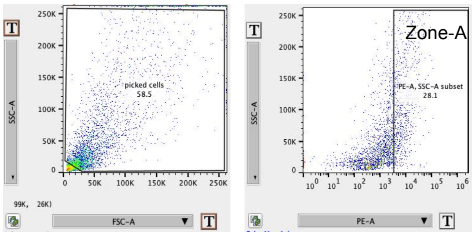

Figure legend 1R: cells in the gate of “selected cells” were selected for further analysis. KI67+ proportion was calculated in Zone-A.

Figure legend 1S: cells in the gate of “selected cells” were selected for further analysis. Apoptosis proportion was calculated in Zone-A and Zone-B.

Figure legend 1T: cells in the gate of “picked cells” were selected for further analysis. CD133+ proportion was calculated in Zone-A.

Gating strategy for **Figure 2K**

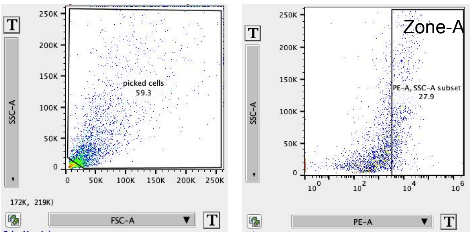

Gating strategy for **Figure 2L**

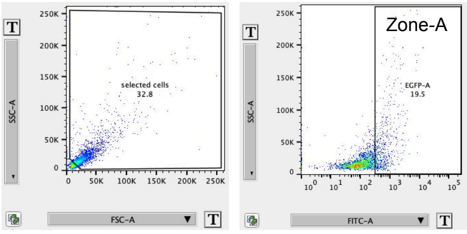

Figure legend 2K: cells in the gate of “picked cells” were selected for further analysis. CD133+ proportion was calculated in Zone-A.

Figure legend 2L: cells in the gate of “selected cells” were selected for further analysis. $LGR5^{EGFP}$+ proportion was calculated in Zone-A.

Gating strategy for **Figure 7D**, **7F**, **7G** and **7K**

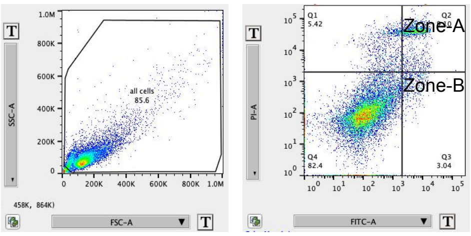

Figure legend 7D, 7F, 7G and 7K: cells in the gate of “all cells” were selected for further analysis. Apoptosis cell proportion was calculated in Zone-A and B.
