# Supplementary material for: Targeting of the m6A eraser ALKBH5 suppresses stemness and chemoresistance of colorectal cancer
Source: Nat Commun. 2025 Dec 13;17:803. doi: 10.1038/s41467-025-67502-0 (PMC12824147; doi:10.1038/s41467-025-67502-0)

S-Figure 1

S-1H

GAPDH

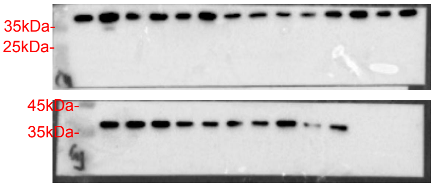

ALKBH5

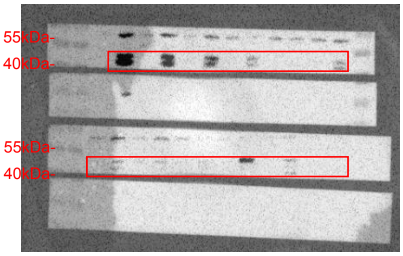

CD133

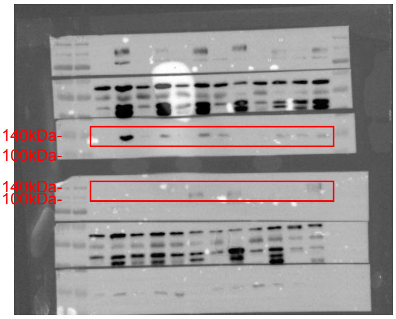

LGR5

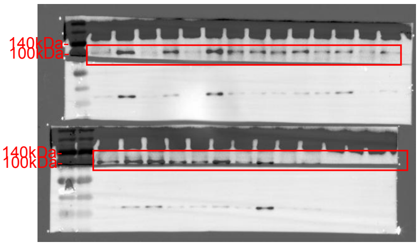

S-Figure 4

S-4A

GAPDH

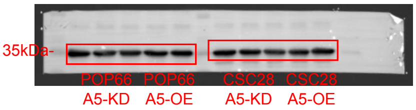

ALKBH5

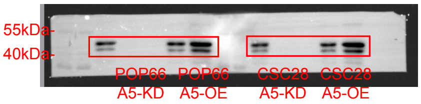

S-Figure 6

S-6A

GAPDH

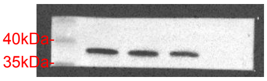

ALKBH5

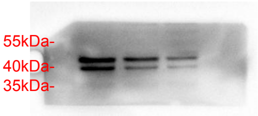

FAM84A

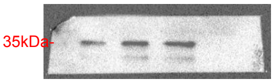

S-6B

GAPDH

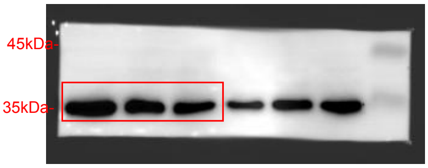

ALKBH5

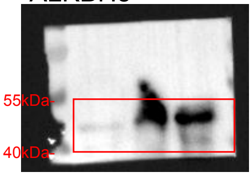

FAM84A

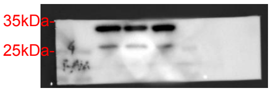

S-6F_POP66

GAPDH

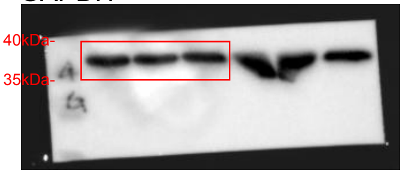

METTL3

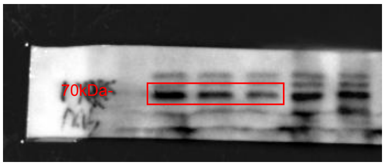

FAM84A

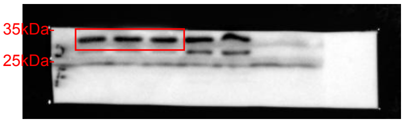

S-Figure 6

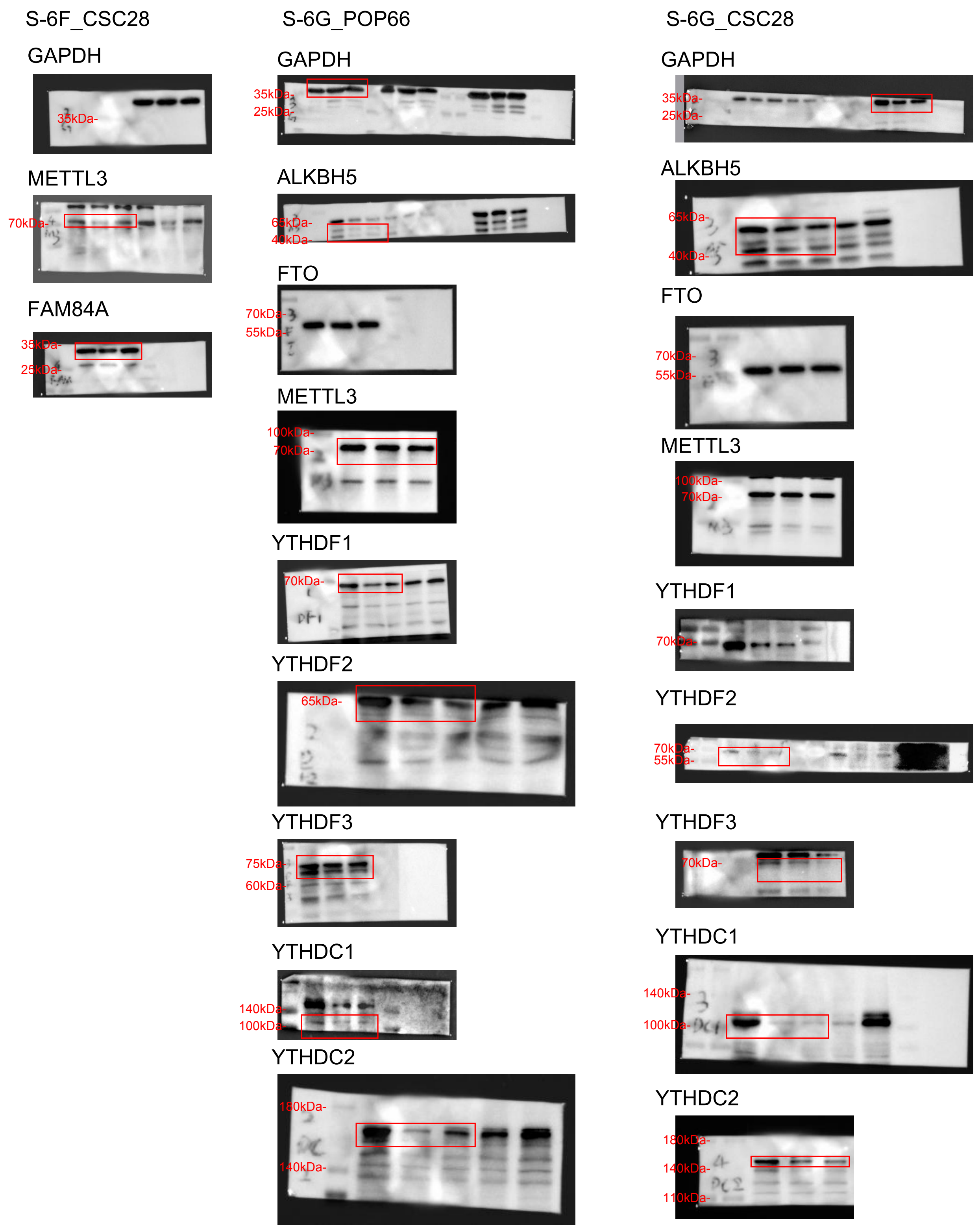
S-6F_CSC28
GAPDH
35kDa-
METTL3
70kDa-
FAM84A
35kDa-
25kDa-
S-6G_POP66
GAPDH
35kDa-
25kDa-
ALKBH5
65kDa-
40kDa-
FTO
70kDa-
55kDa-
METTL3
100kDa-
70kDa-
YTHDF1
70kDa-
YTHDF2
65kDa-
YTHDF3
75kDa-
60kDa-
YTHDC1
140kDa-
100kDa-
YTHDC2
180kDa-
140kDa-
S-6G_CSC28
GAPDH
35kDa-
25kDa-
ALKBH5
65kDa-
40kDa-
FTO
70kDa-
55kDa-
METTL3
100kDa-
70kDa-
YTHDF1
70kDa-
YTHDF2
70kDa-
55kDa-
YTHDF3
70kDa-
YTHDC1
140kDa-
100kDa-
YTHDC2
180kDa-
140kDa-
110kDa-

S-Figure 6

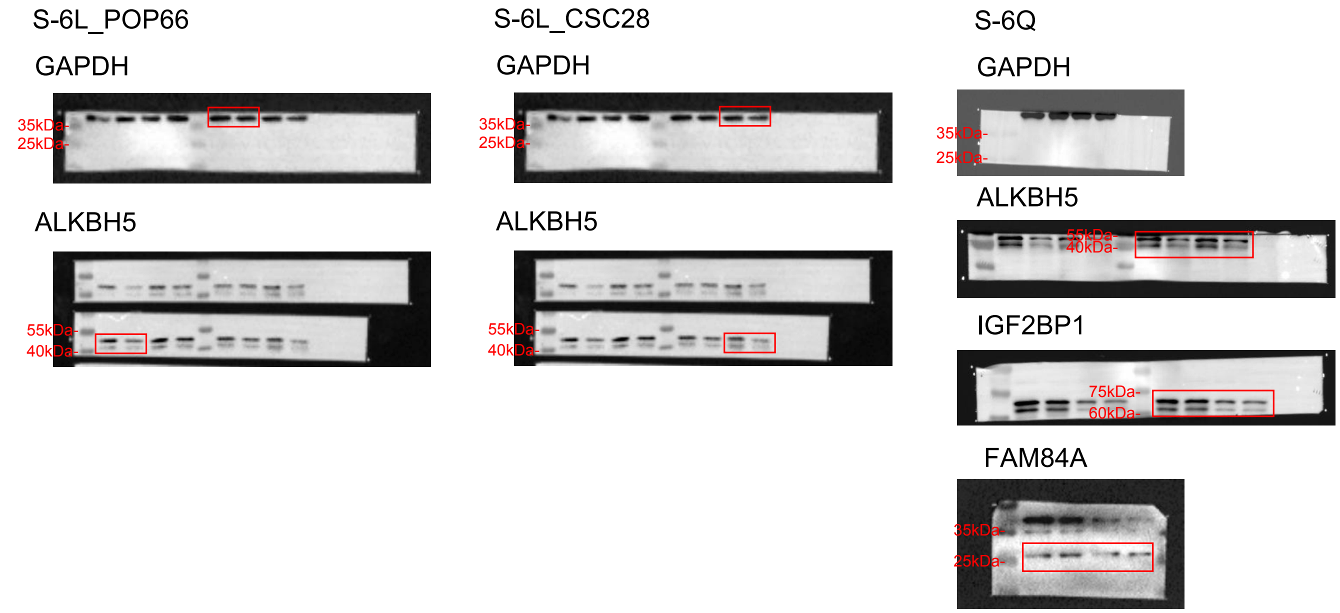

S-Figure 7

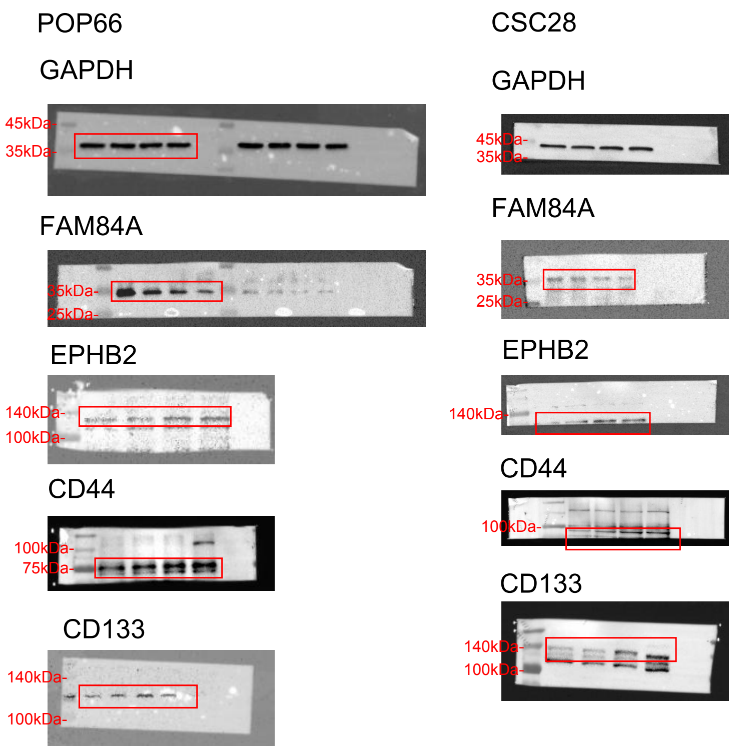

S-Figure 9

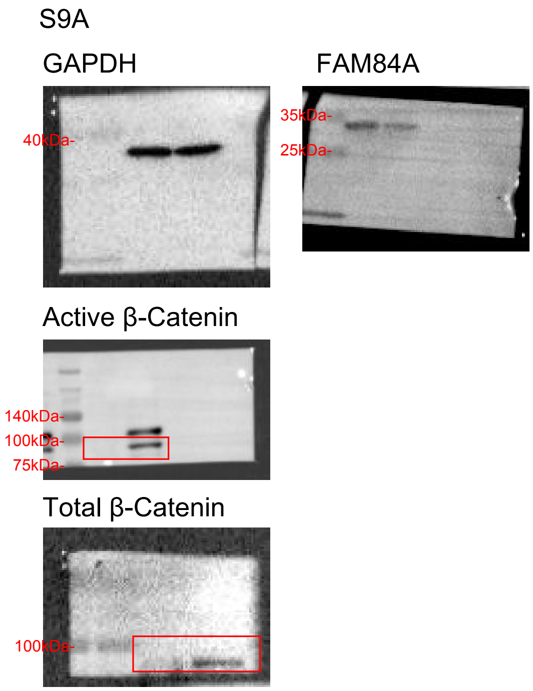

S-Figure 9

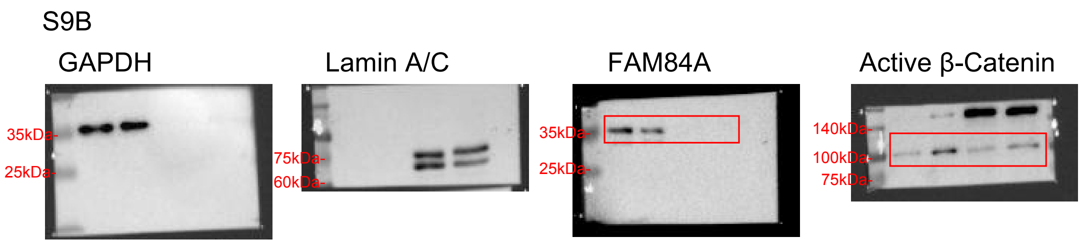

S-Figure 9

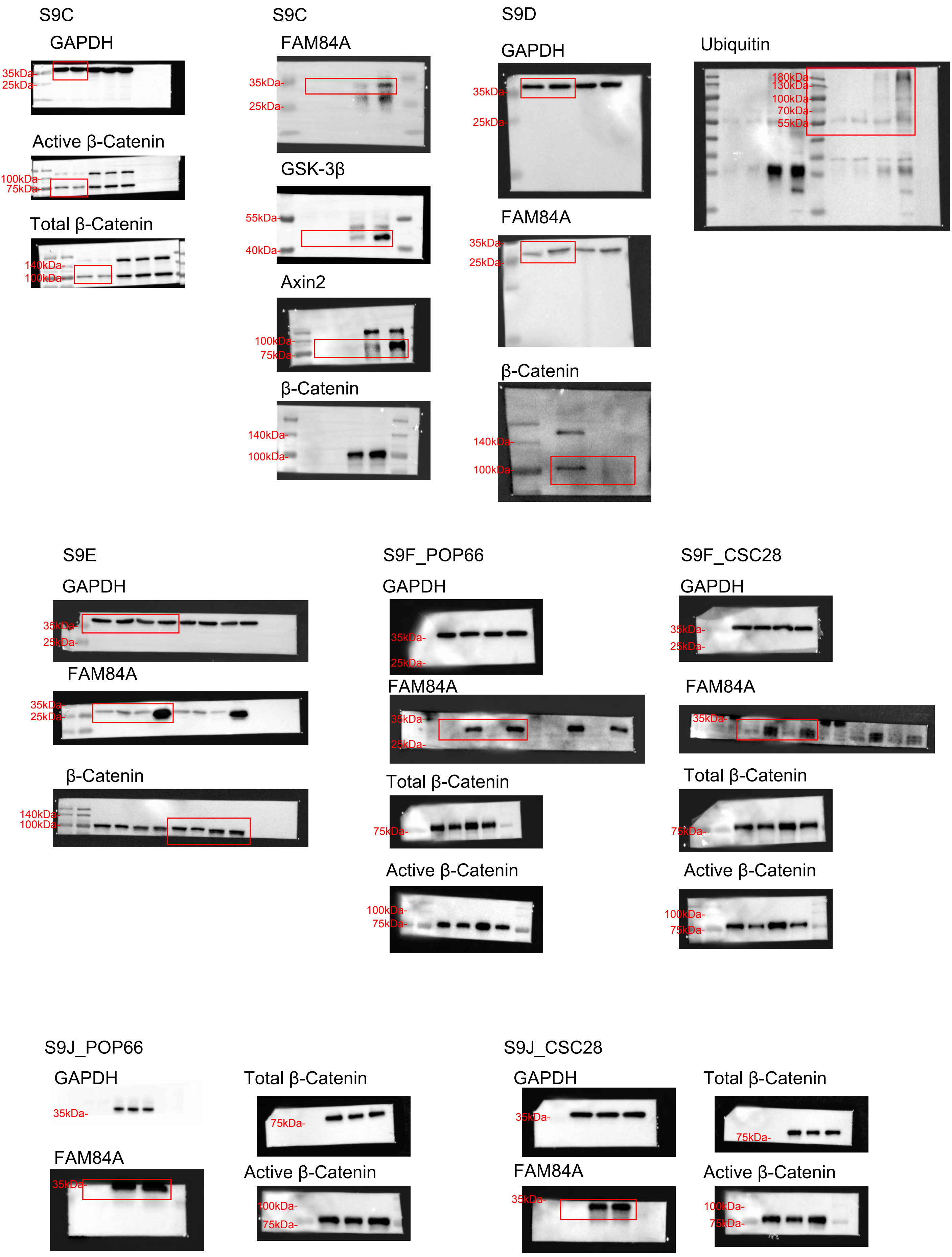
S9C
GAPDH
35kDa-
25kDa-
Active β-Catenin
100kDa-
75kDa-
Total β-Catenin
140kDa-
100kDa-
S9C
FAM84A
35kDa-
25kDa-
GSK-3β
55kDa-
40kDa-
Axin2
100kDa-
75kDa-
β-Catenin
140kDa-
100kDa-
S9D
GAPDH
35kDa-
25kDa-
FAM84A
35kDa-
25kDa-
β-Catenin
140kDa-
100kDa-
Ubiquitin
180kDa-
130kDa-
100kDa-
70kDa-
55kDa-
S9E
GAPDH
35kDa-
25kDa-
FAM84A
35kDa-
25kDa-
β-Catenin
140kDa-
100kDa-
S9F_POP66
GAPDH
35kDa-
25kDa-
FAM84A
35kDa-
25kDa-
Total β-Catenin
75kDa-
Active β-Catenin
100kDa-
75kDa-
S9F_CSC28
GAPDH
35kDa-
25kDa-
FAM84A
35kDa-
Total β-Catenin
75kDa-
Active β-Catenin
100kDa-
75kDa-
S9J_POP66
GAPDH
35kDa-
FAM84A
35kDa-
Total β-Catenin
75kDa-
Active β-Catenin
100kDa-
75kDa-
S9J_CSC28
GAPDH
35kDa-
FAM84A
35kDa-
Total β-Catenin
75kDa-
Active β-Catenin
100kDa-
75kDa-

S-Figure 13

S13A_POP66

GAPDH

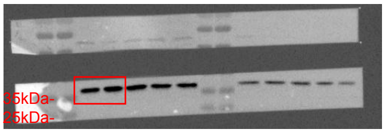

ALKBH5

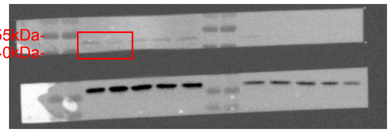

S13A_CSC28

GAPDH

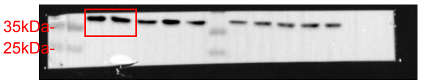

ALKBH5

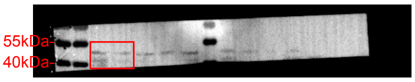

Supplement: Supplementary file 7 — Source Data [file 41467_2025_67502_MOESM7_ESM.zip › Source Data/Supplementary information_ Raw data for western blot inS-Figures.pdf]
