# Supplementary material for: Targeting of the m6A eraser ALKBH5 suppresses stemness and chemoresistance of colorectal cancer
Source: Nat Commun. 2025 Dec 13;17:803. doi: 10.1038/s41467-025-67502-0 (PMC12824147; doi:10.1038/s41467-025-67502-0)
Supplement: Supplementary file 7 — Source Data [file 41467_2025_67502_MOESM7_ESM.zip › Source Data/Supplementary information_Raw data for western blot in main Figures.pdf]

Figure 1

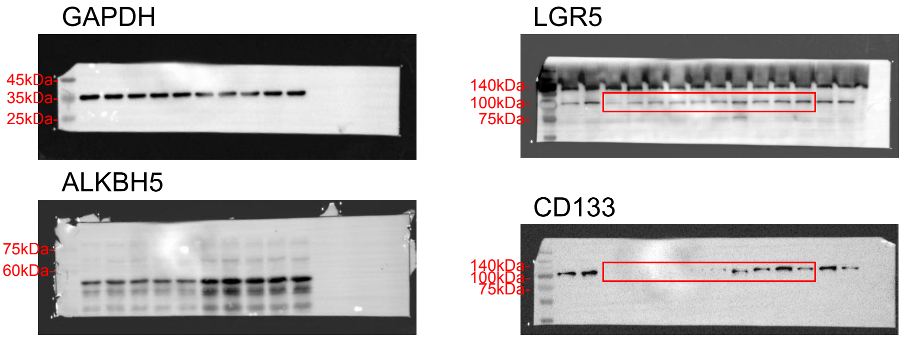
GAPDH
45kDa
35kDa
25kDa
LGR5
140kDa
100kDa
75kDa
ALKBH5
75kDa
60kDa
CD133
140kDa
100kDa
75kDa

Figure 2

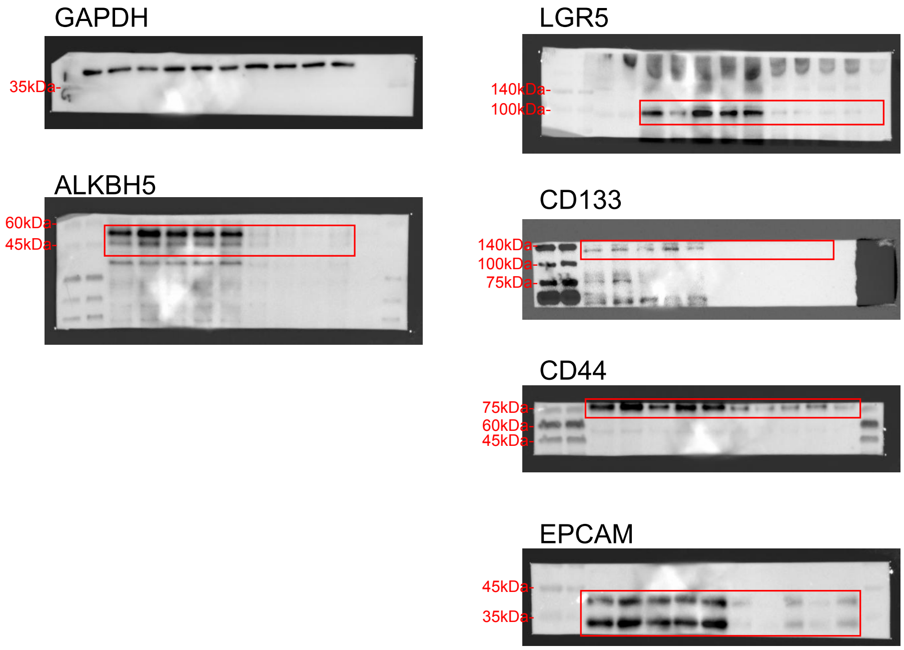
GAPDH
35kDa-
LGR5
140kDa-
100kDa-
ALKBH5
60kDa-
45kDa-
CD133
140kDa-
100kDa-
75kDa-
CD44
75kDa-
60kDa-
45kDa-
EPCAM
45kDa-
35kDa-

Figure 3

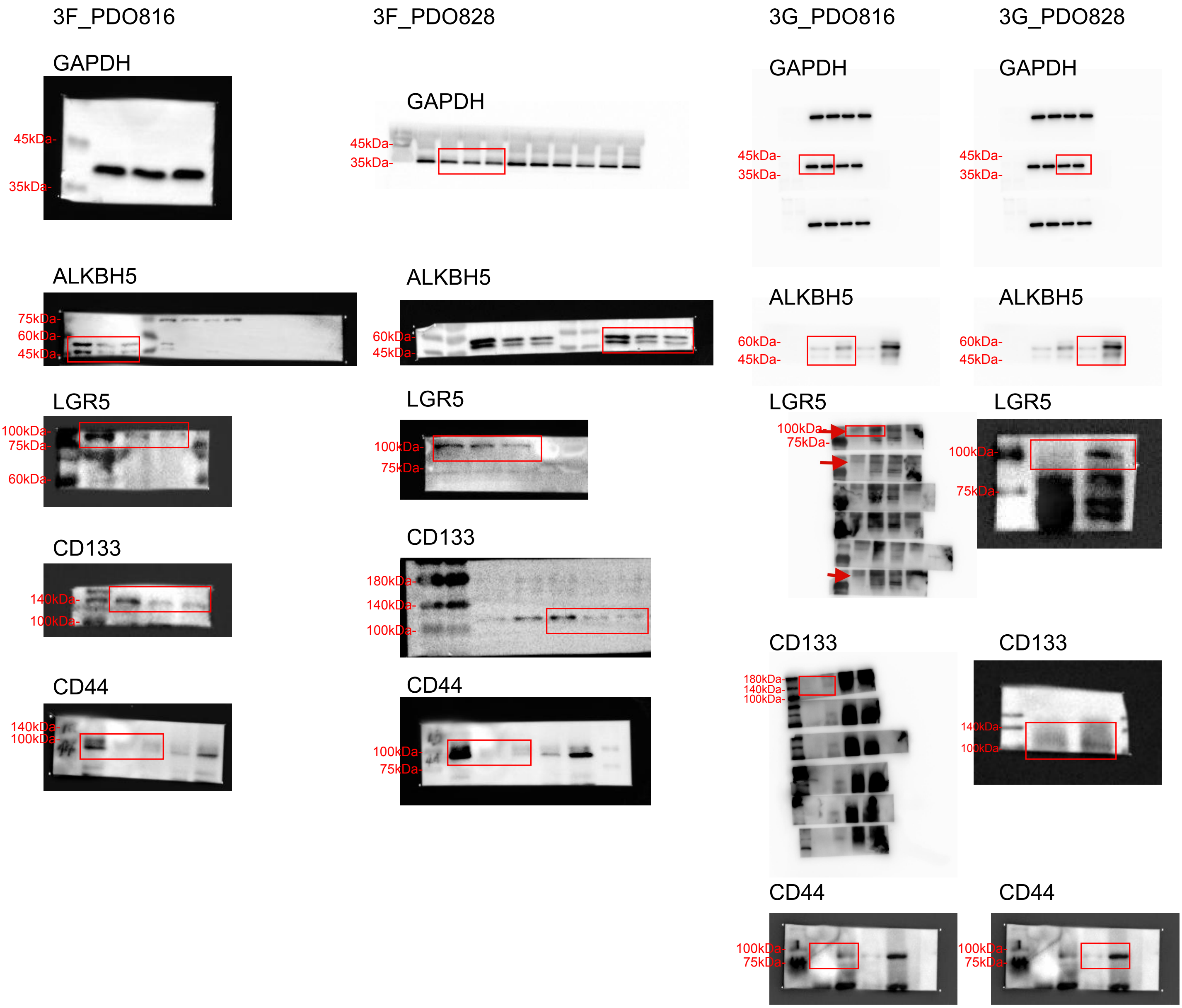
3F_PDO816
3F_PDO828
3G_PDO816
3G_PDO828
GAPDH
45kDa-
35kDa-
ALKBH5
75kDa-
60kDa-
45kDa-
LGR5
100kDa-
75kDa-
60kDa-
CD133
180kDa-
140kDa-
100kDa-
CD44

Figure 3

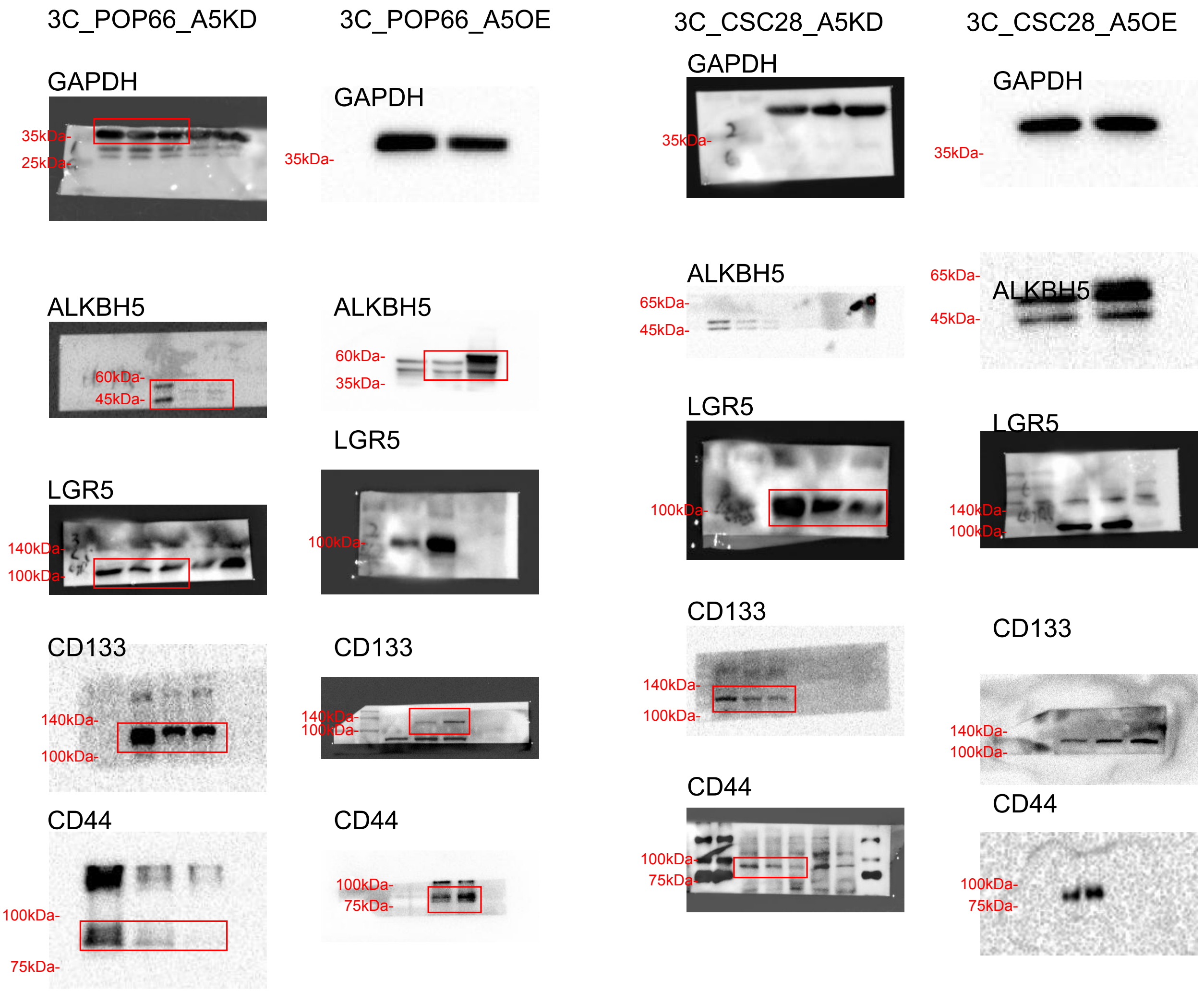
3C_POP66_A5KD
3C_POP66_A5OE
3C_CSC28_A5KD
3C_CSC28_A5OE
GAPDH
35kDa-
25kDa-
ALKBH5
60kDa-
45kDa-
35kDa-
65kDa-
LGR5
140kDa-
100kDa-
CD133
CD44
75kDa-

Figure 4

4A_CSC28-A5KD

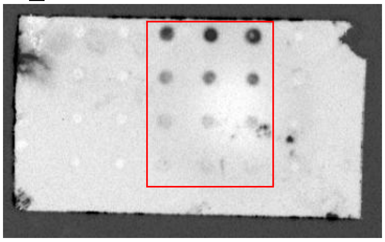

4A_CSC28-A5OE

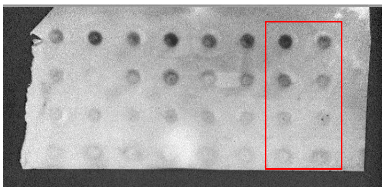

4M_POP66

GAPDH

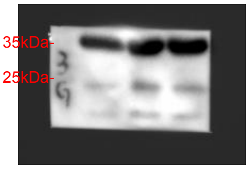

4M_CSC28

GAPDH

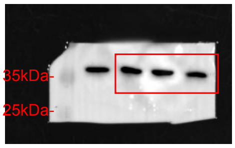

ALKBH5

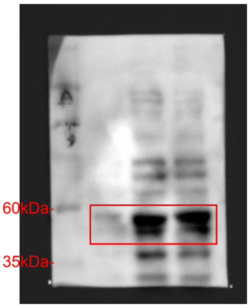

ALKBH5

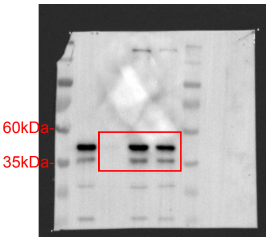

dCas-ALKBH5 fusion

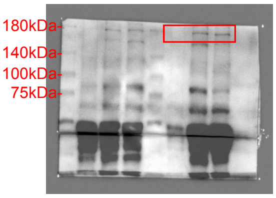

dCas-ALKBH5 fusion

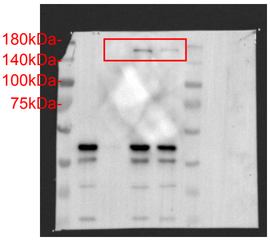

Figure 5

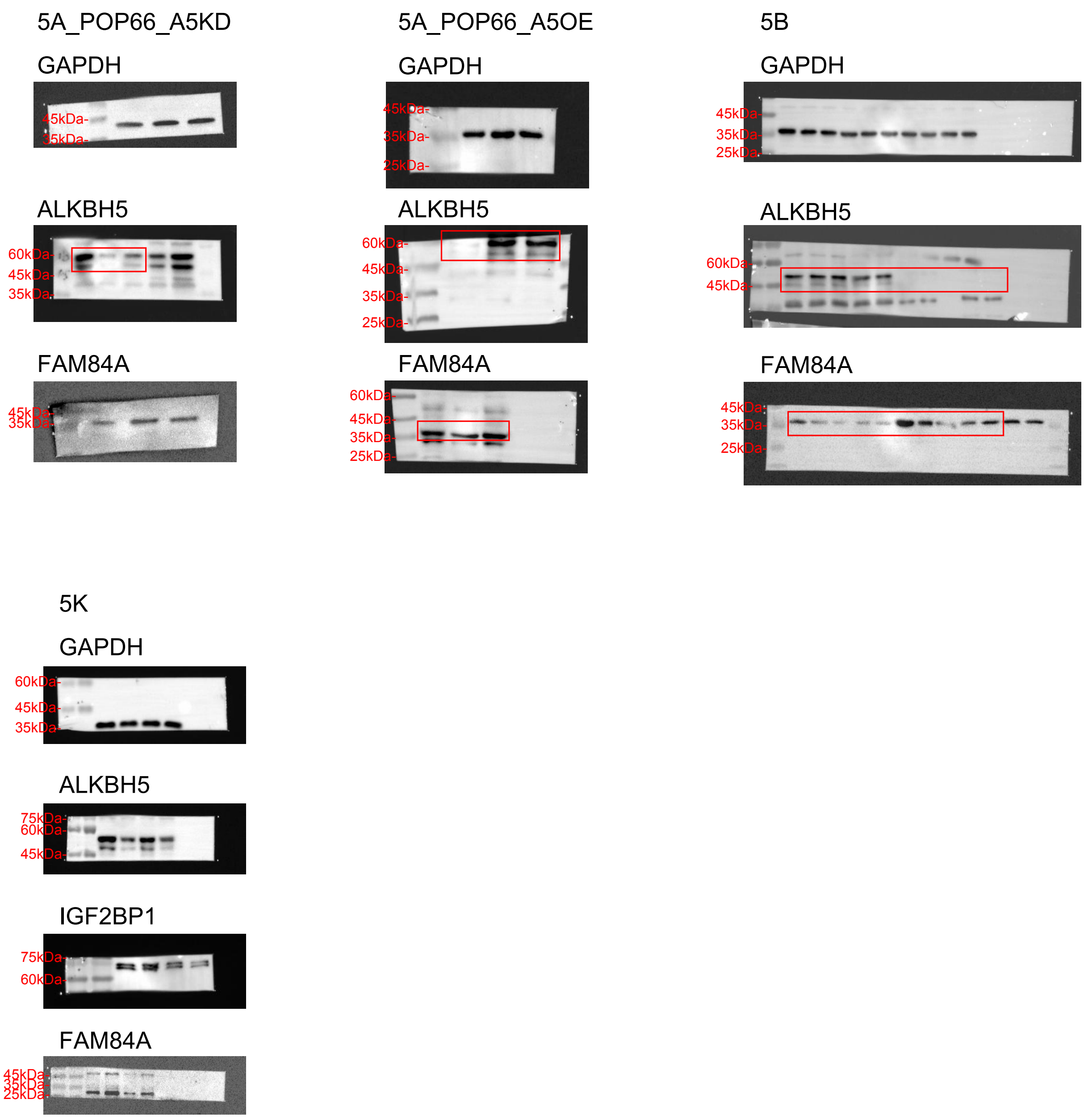
5A_POP66_A5KD
GAPDH
45kDa-
35kDa-
ALKBH5
60kDa-
45kDa-
35kDa-
FAM84A
45kDa-
35kDa-
5A_POP66_A5OE
GAPDH
45kDa-
35kDa-
25kDa-
ALKBH5
60kDa-
45kDa-
35kDa-
25kDa-
FAM84A
60kDa-
45kDa-
35kDa-
25kDa-
5B
GAPDH
45kDa-
35kDa-
25kDa-
ALKBH5
60kDa-
45kDa-
FAM84A
45kDa-
35kDa-
25kDa-
5K
GAPDH
60kDa-
45kDa-
35kDa-
ALKBH5
75kDa-
60kDa-
45kDa-
IGF2BP1
75kDa-
60kDa-
FAM84A
45kDa-
35kDa-
25kDa-

Figure 6

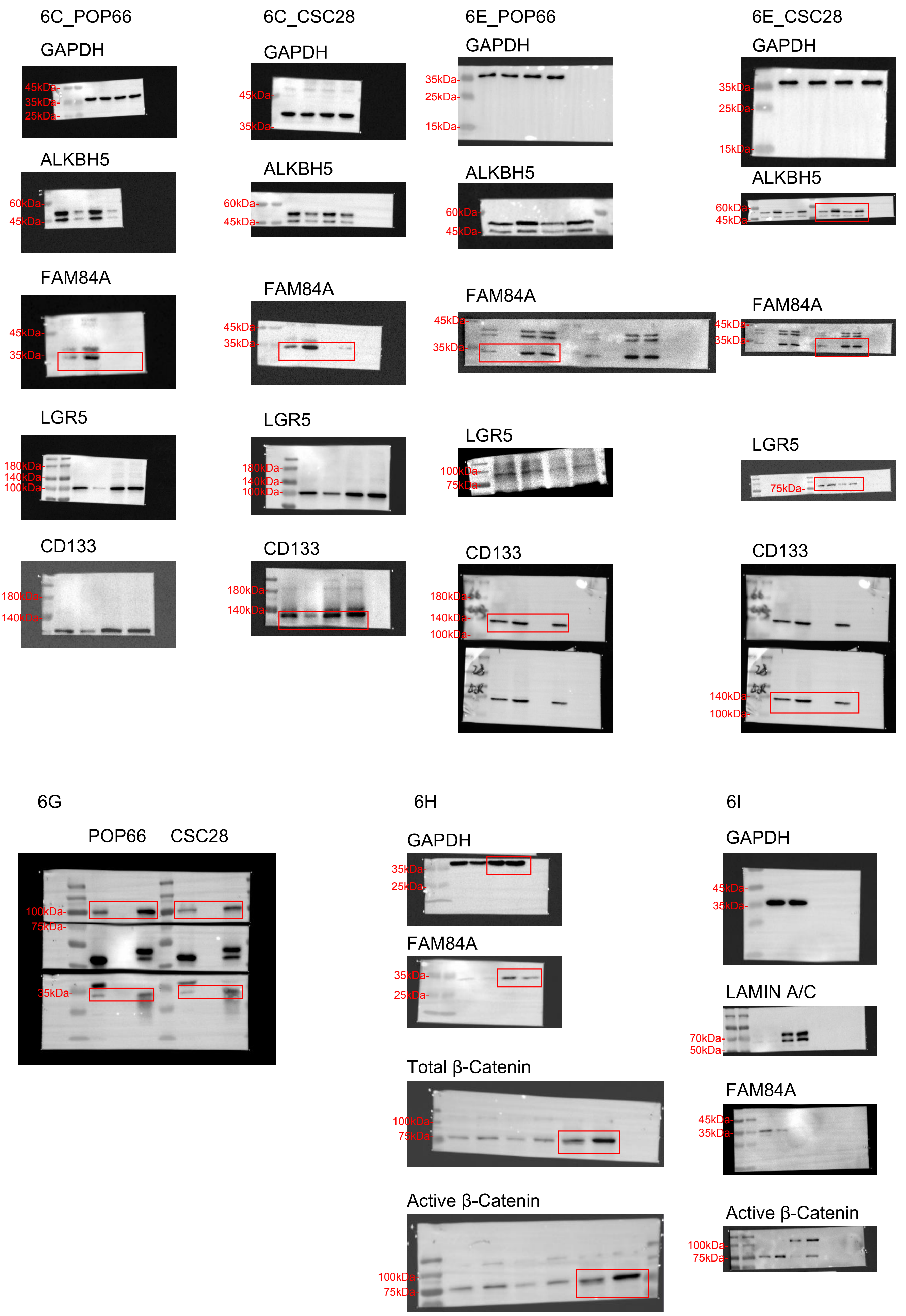
6C_POP66
GAPDH
45kDa-
35kDa-
25kDa-
ALKBH5
60kDa-
45kDa-
FAM84A
45kDa-
35kDa-
LGR5
180kDa-
140kDa-
100kDa-
CD133
180kDa-
140kDa-
6C_CSC28
GAPDH
45kDa-
35kDa-
ALKBH5
60kDa-
45kDa-
FAM84A
45kDa-
35kDa-
LGR5
180kDa-
140kDa-
100kDa-
CD133
180kDa-
140kDa-
6E_POP66
GAPDH
35kDa-
25kDa-
15kDa-
ALKBH5
60kDa-
45kDa-
FAM84A
45kDa-
35kDa-
LGR5
100kDa-
75kDa-
CD133
180kDa-
140kDa-
100kDa-
6E_CSC28
GAPDH
35kDa-
25kDa-
15kDa-
ALKBH5
60kDa-
45kDa-
FAM84A
45kDa-
35kDa-
LGR5
75kDa-
CD133
140kDa-
100kDa-
6G
POP66
CSC28
100kDa-
75kDa-
35kDa-
6H
GAPDH
35kDa-
25kDa-
FAM84A
35kDa-
25kDa-
Total β-Catenin
100kDa-
75kDa-
Active β-Catenin
100kDa-
75kDa-
6I
GAPDH
45kDa-
35kDa-
LAMIN A/C
70kDa-
50kDa-
FAM84A
45kDa-
35kDa-
Active β-Catenin
100kDa-
75kDa-

Figure 6

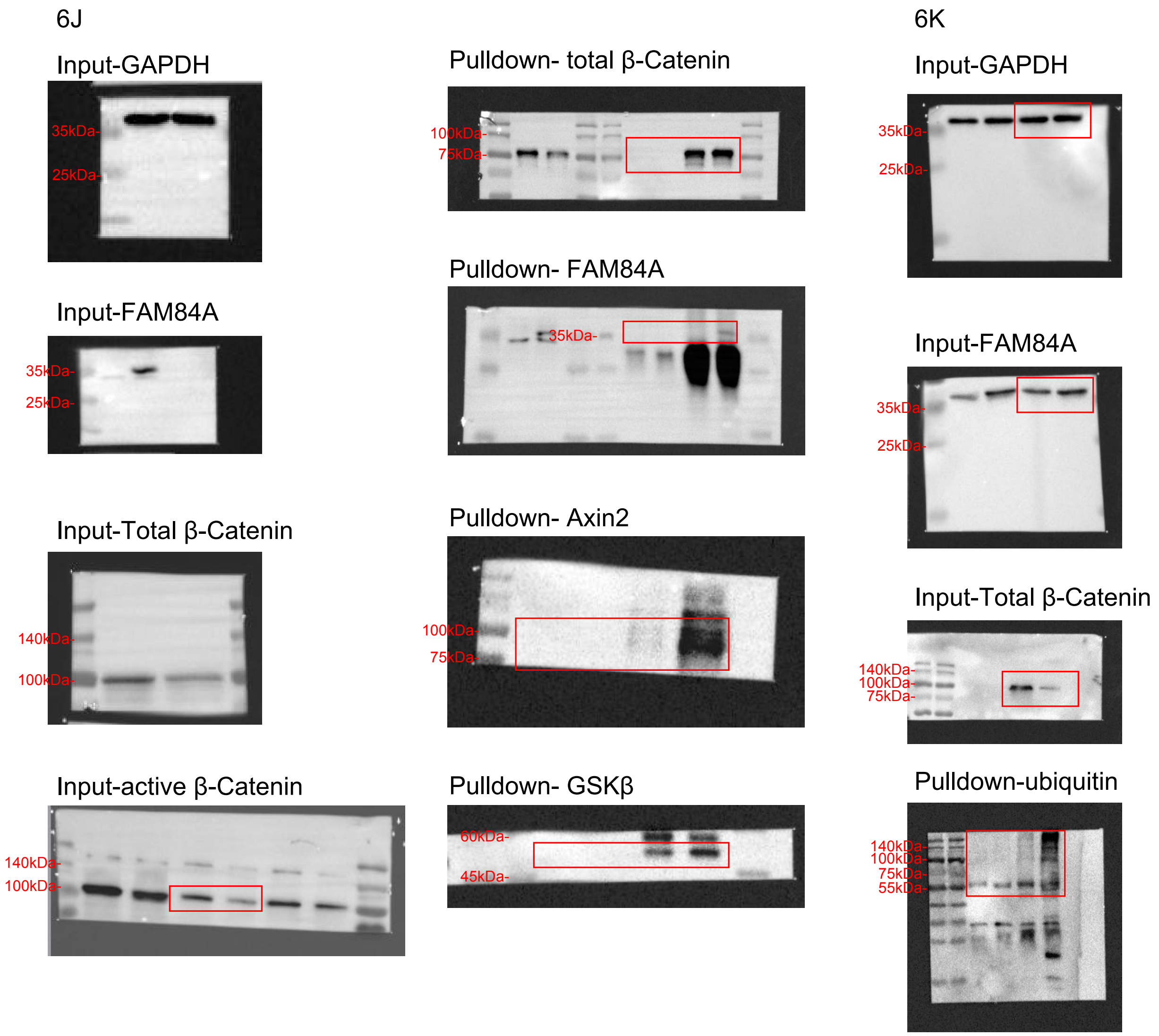
6J
Input-GAPDH
35kDa-
25kDa-
Input-FAM84A
35kDa-
25kDa-
Input-Total β-Catenin
140kDa-
100kDa-
Input-active β-Catenin
140kDa-
100kDa-
Pulldown- total β-Catenin
100kDa-
75kDa-
Pulldown- FAM84A
35kDa-
Pulldown- Axin2
100kDa-
75kDa-
Pulldown- GSKβ
60kDa-
45kDa-
6K
Input-GAPDH
35kDa-
25kDa-
Input-FAM84A
35kDa-
25kDa-
Input-Total β-Catenin
140kDa-
100kDa-
75kDa-
Pulldown-ubiquitin
140kDa-
100kDa-
75kDa-
55kDa-

Figure 6

6L

GAPDH

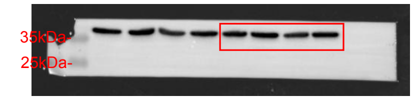

FAM84A

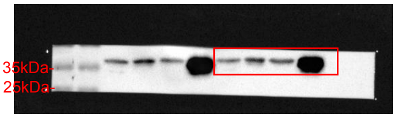

Total β-Catenin

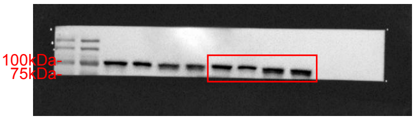

6M

POP66_GAPDH

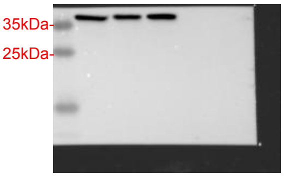

POP66-FAM84A

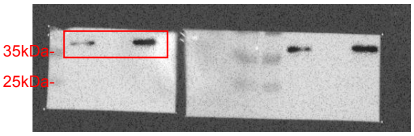

POP66-Total β-Catenin

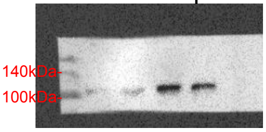

POP66-ALKBH5

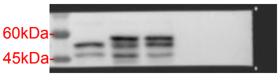

CSC28_GAPDH

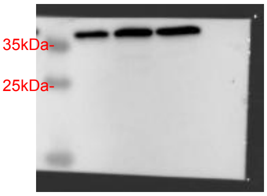

CSC28-FAM84A

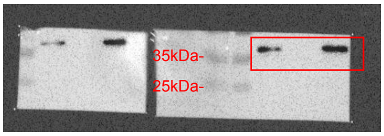

CSC28-Total β-Catenin

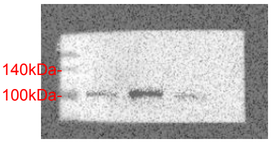

CSC28-ALKBH5

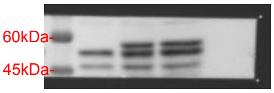

6N

POP66_input-GAPDH

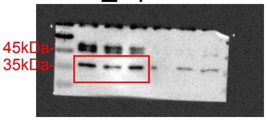

CSC28_input-GAPDH

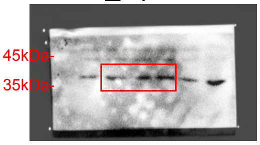

POP66-input-Total β-Catenin

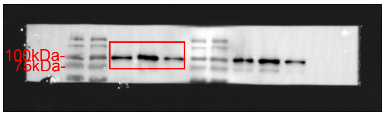

CSC28-input-Total β-Catenin

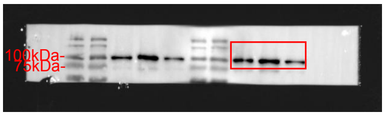

POP66-pulldown-ubiquitin

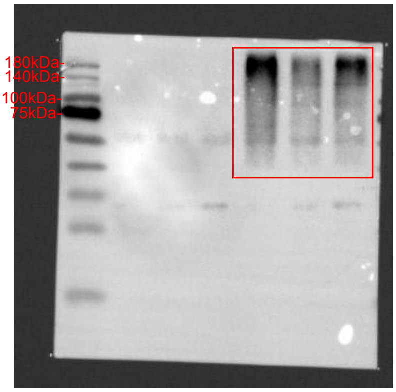

CSC28-pulldown-ubiquitin

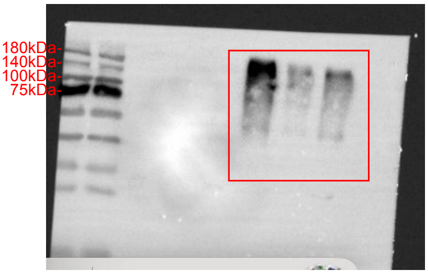

6O

GAPDH

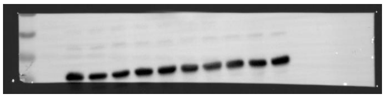

ALKBH5

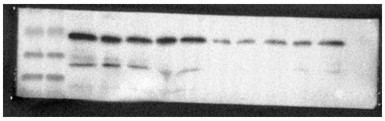

Total β-Catenin

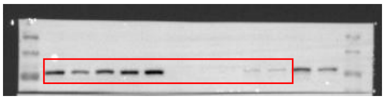

Active β-Catenin

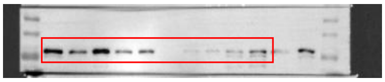

Figure 7

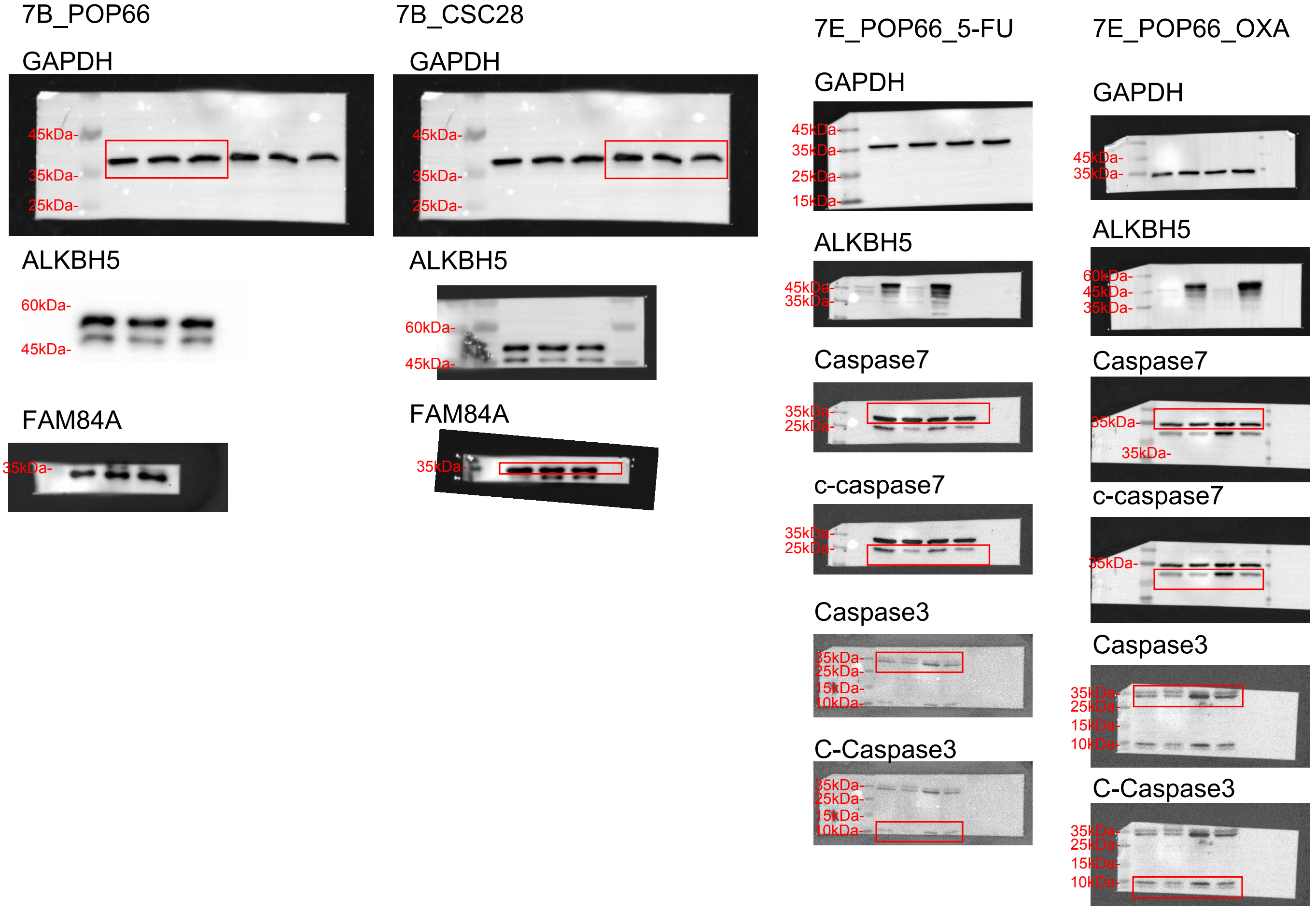
7B_POP66
GAPDH
45kDa-
35kDa-
25kDa-
ALKBH5
60kDa-
45kDa-
FAM84A
35kDa-
7B_CSC28
GAPDH
45kDa-
35kDa-
25kDa-
ALKBH5
60kDa-
45kDa-
FAM84A
35kDa-
7E_POP66_5-FU
GAPDH
45kDa-
35kDa-
25kDa-
15kDa-
ALKBH5
45kDa-
35kDa-
Caspase7
35kDa-
25kDa-
c-caspase7
35kDa-
25kDa-
Caspase3
35kDa-
25kDa-
15kDa-
10kDa-
C-Caspase3
35kDa-
25kDa-
15kDa-
10kDa-
7E_POP66_OXA
GAPDH
45kDa-
35kDa-
ALKBH5
60kDa-
45kDa-
35kDa-
Caspase7
35kDa-
35kDa-
c-caspase7
35kDa-
Caspase3
35kDa-
25kDa-
15kDa-
10kDa-
C-Caspase3
35kDa-
25kDa-
15kDa-
10kDa-

Figure 7

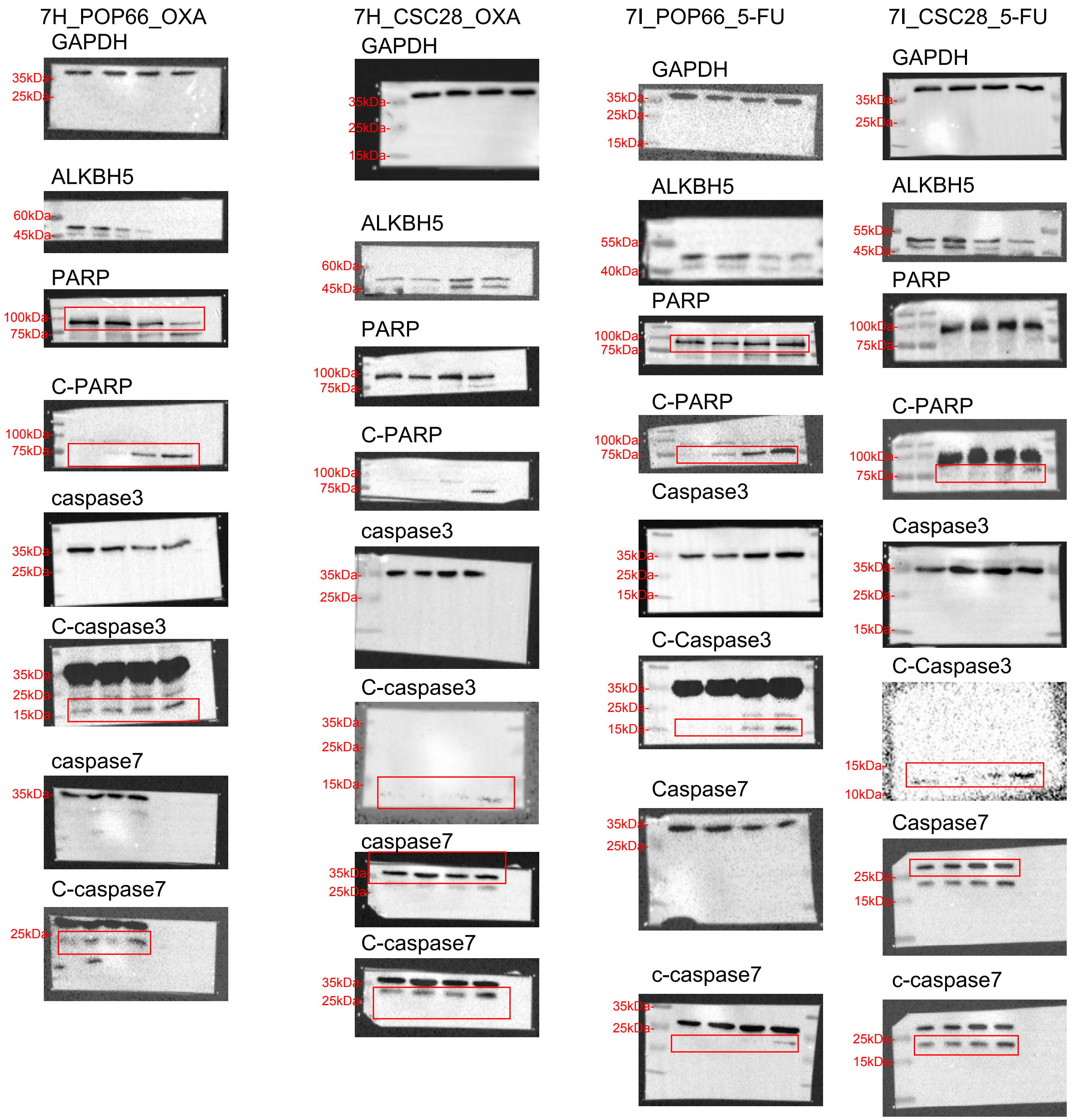
7H_POP66_OXA
GAPDH
35kDa-
25kDa-
ALKBH5
60kDa-
45kDa-
PARP
100kDa-
75kDa-
C-PARP
100kDa-
75kDa-
caspase3
35kDa-
25kDa-
C-caspase3
35kDa-
25kDa-
15kDa-
caspase7
35kDa-
C-caspase7
25kDa-
7H_CSC28_OXA
GAPDH
35kDa-
25kDa-
15kDa-
ALKBH5
60kDa-
45kDa-
PARP
100kDa-
75kDa-
C-PARP
100kDa-
75kDa-
caspase3
35kDa-
25kDa-
C-caspase3
35kDa-
25kDa-
15kDa-
caspase7
35kDa-
25kDa-
C-caspase7
35kDa-
25kDa-
7I_POP66_5-FU
GAPDH
35kDa-
25kDa-
15kDa-
ALKBH5
55kDa-
40kDa-
PARP
100kDa-
75kDa-
C-PARP
100kDa-
75kDa-
Caspase3
35kDa-
25kDa-
15kDa-
C-Caspase3
35kDa-
25kDa-
15kDa-
Caspase7
35kDa-
25kDa-
c-caspase7
35kDa-
25kDa-
7I_CSC28_5-FU
GAPDH
35kDa-
25kDa-
ALKBH5
55kDa-
45kDa-
PARP
100kDa-
75kDa-
C-PARP
100kDa-
75kDa-
Caspase3
35kDa-
25kDa-
15kDa-
C-Caspase3
15kDa-
10kDa-
Caspase7
25kDa-
15kDa-
c-caspase7
25kDa-
15kDa-

Figure 8

8E_cKO+5-FU

GAPDH

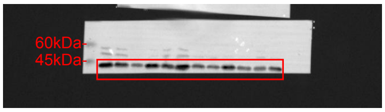

ALKBH5

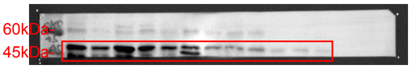

PARP

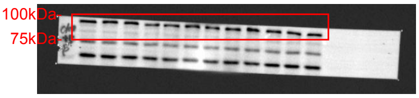

C-PARP

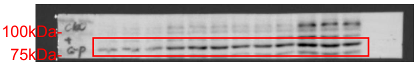

Caspase3

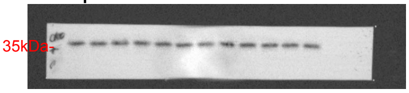

C-Caspase3

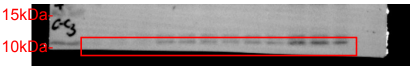

Caspase7

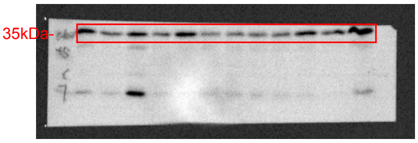

C-Caspase7

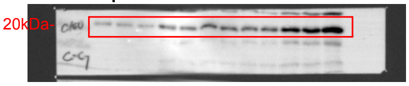

8E_cKO+OXA

GAPDH

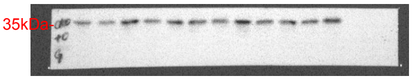

ALKBH5

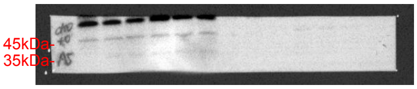

PARP

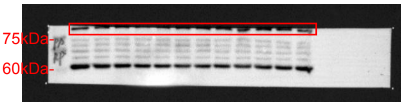

C-PARP

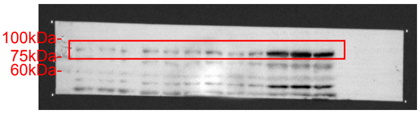

Caspase3

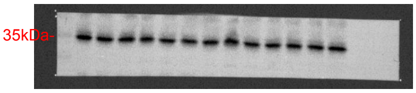

C-Caspase3

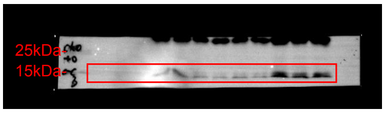

Caspase7

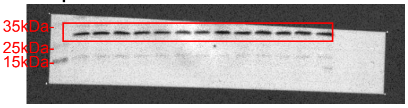

C-Caspase7

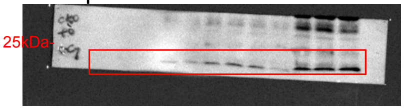

Figure 8

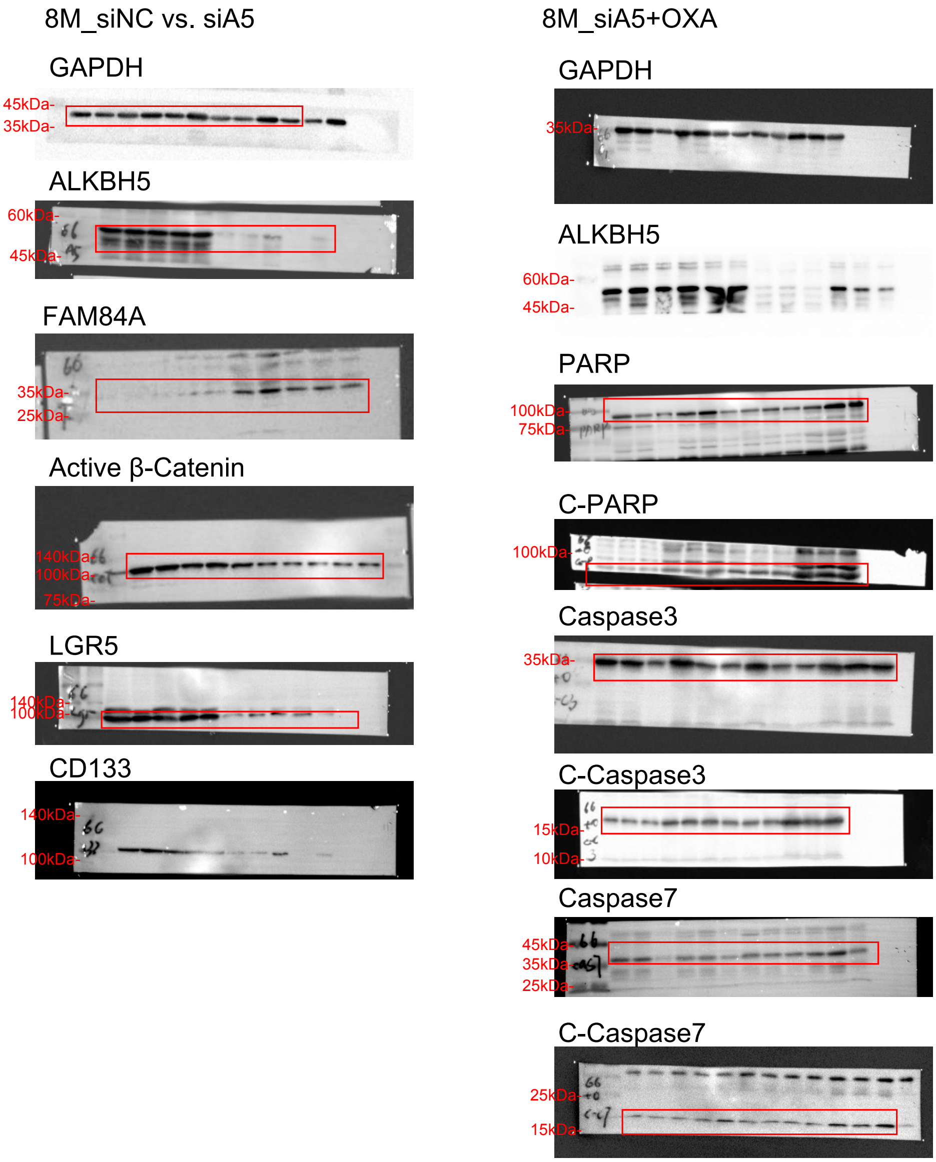
8M_siNC vs. siA5
GAPDH
45kDa-
35kDa-
ALKBH5
60kDa-
45kDa-
FAM84A
35kDa-
25kDa-
Active β-Catenin
140kDa-
100kDa-
75kDa-
LGR5
140kDa-
100kDa-
CD133
140kDa-
100kDa-
8M_siA5+OXA
GAPDH
35kDa-
ALKBH5
60kDa-
45kDa-
PARP
100kDa-
75kDa-
C-PARP
100kDa-
Caspase3
35kDa-
C-Caspase3
15kDa-
10kDa-
Caspase7
45kDa-
35kDa-
25kDa-
C-Caspase7
25kDa-
15kDa-

Figure 8

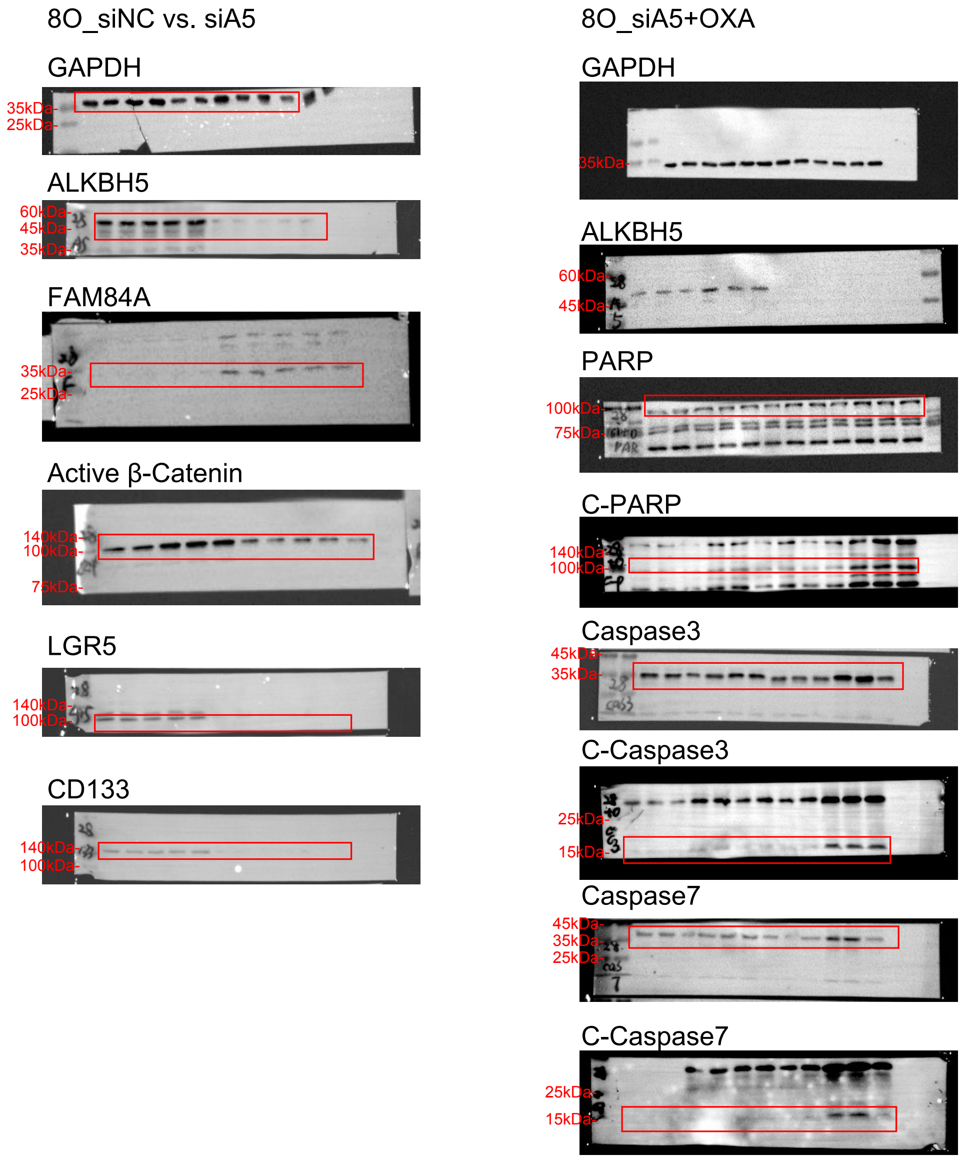
8O_siNC vs. siA5
GAPDH
35kDa-
25kDa-
ALKBH5
60kDa-
45kDa-
35kDa-
FAM84A
35kDa-
25kDa-
Active β-Catenin
140kDa-
100kDa-
75kDa-
LGR5
140kDa-
100kDa-
CD133
140kDa-
100kDa-
8O_siA5+OXA
GAPDH
35kDa-
ALKBH5
60kDa-
45kDa-
PARP
100kDa-
75kDa-
C-PARP
140kDa-
100kDa-
Caspase3
45kDa-
35kDa-
C-Caspase3
25kDa-
15kDa-
Caspase7
45kDa-
35kDa-
25kDa-
C-Caspase7
25kDa-
15kDa-
